# Optimized diagnosis-based comorbidity measures for all-cause mortality prediction in a national population-based ICU population

**DOI:** 10.1186/s13054-022-04172-0

**Published:** 2022-10-06

**Authors:** Anna Aronsson Dannewitz, Bodil Svennblad, Karl Michaëlsson, Miklos Lipcsey, Rolf Gedeborg

**Affiliations:** Department of Surgical Sciences, Uppsala University, Uppsala, Sweden

**Keywords:** Critical care, Intensive care, Comorbidity, Mortality, Readmission, Simplified Acute Physiology Score

## Abstract

**Background:**

We aimed to optimize prediction of long-term all-cause mortality of intensive care unit (ICU) patients, using quantitative register-based comorbidity information assessed from hospital discharge diagnoses prior to intensive care treatment.

**Material and methods:**

Adult ICU admissions during 2006 to 2012 in the Swedish intensive care register were followed for at least 4 years. The performance of quantitative comorbidity measures based on the 5-year history of number of hospital admissions, length of stay, and time since latest admission in 36 comorbidity categories was compared in time-to-event analyses with the Charlson comorbidity index (CCI) and the Simplified Acute Physiology Score (SAPS3).

**Results:**

During a 7-year period, there were 230,056 ICU admissions and 62,225 deaths among 188,965 unique individuals. The time interval from the most recent hospital stays and total length of stay within each comorbidity category optimized mortality prediction and provided clear separation of risk categories also within strata of age and CCI, with hazard ratios (HRs) comparing lowest to highest quartile ranging from 1.17 (95% CI: 0.52–2.64) to 6.41 (95% CI: 5.19–7.92). Risk separation was also observed within SAPS deciles with HR ranging from 1.07 (95% CI: 0.83–1.38) to 3.58 (95% CI: 2.12–6.03).

**Conclusion:**

Baseline comorbidity measures that included the time interval from the most recent hospital stay in 36 different comorbidity categories substantially improved long-term mortality prediction after ICU admission compared to the Charlson index and the SAPS score.

*Trial registration* ClinicalTrials.gov ID NCT04109001, date of registration 2019-09-26 retrospectively.

**Supplementary Information:**

The online version contains supplementary material available at 10.1186/s13054-022-04172-0.

## Introduction

In outcomes research on general intensive care unit (ICU) populations, accurate measures of comorbidity are needed to predict mortality, to control confounding, and to define relevant population strata [1–3].

Weighted comorbid conditions identified in general health-care registers can contribute to ICU risk adjustment models [4]. The widely used Simplified Acute Physiology Score (SAPS3) includes some information on comorbidity [5, 6]. The Charlson comorbidity index (CCI) is a widely used general comorbidity measure [1, 7] but may not be sufficiently discriminative [8]. A common feature of these risk scores is that they only use the presence of comorbidity as binary variables and consequently disregard potential quantitative information [1, 7, 9, 10]. A previous Danish study demonstrated that the CCI was combined with other readily available administrative data performed as well as physiology-based scoring systems in predicting mortality [7].

We hypothesized that the information extracted from previous hospital admissions could be optimized by expanding the number of comorbidity categories and extracting quantitative information for each category. The aim of this study was to improve prediction of long-term mortality rate after an ICU stay, using increased granularity of hospital discharge history while considering the impact of the length of follow-up and the potential bias introduced by readmission as a competing event.

## Methods

### Study population

The study population included all patients > 15 years old admitted to an ICU during the years 2006 to 2012 and registered in the Swedish Intensive Care Registry (SIR) [11] In 2012, SIR covered 92% of ICU admissions in Sweden, representing all types of ICUs. Follow-up started on the ICU admission date. ICU admissions separated by less than 24 h representing transfers between units or early readmissions were joined retaining covariates from the first admission to the merged admission.

Hospital discharge diagnoses from in-patient care five years preceding the index time for the ICU admission were extracted from the National Patient Register (NPR) and linked to ICU admissions using unique person identity numbers [12, 13] The regional Human Ethics Committee approved the study (Approval no 2012/197).

### Exposures

The number of previous ICU stays in the 365 days preceding ICU admission, the time since the most recent previous ICU stay (1–7 days, 8–30 days, 31–90 days, 91–365 days, > 365 days), and the total length of previous ICU stays were used as covariates in the most basic prediction model together with age and sex.

The CCI was calculated from hospital discharge diagnoses during five years prior to start of follow-up [3]. For each of 36 comorbidity categories (see Additional file 1: eTable S1), the number of admissions with a primary diagnosis, the number of admissions with a secondary diagnosis, the total length of stay with a primary diagnosis, and the interval from the last admission with the comorbidity condition as a primary diagnosis (0–1 month, 1–6 months, 6–12 months, 1–3 years, > 3 years) were calculated. The SAPS version 3 was available in SIR [5, 6].

### Outcomes

Follow-up for all-cause mortality was available until December 31, 2016. ICU readmission and a composite outcome of death and ICU readmission were analyzed as secondary outcomes.

### Statistical methods

The data were split into a training (the first 5 years of the study period) and a validation (the last 2 years) dataset. In a subgroup analysis, the validation data were restricted to ICU admissions where the patient had previous hospitalizations with main diagnoses representing at least two different comorbidity categories.

Cox proportional hazard models were estimated on the training dataset with a robust sandwich estimator to handle the dependency in patients with multiple admissions [14]. Visual inspection of generalized additive model (GAM) plots for death in the training dataset was used to verify that age and length of previous ICU admissions could be modeled as linear effects (Additional file 1: eFigs. S1 and S2).

Because of non-nested models, the Akaike information criterion (AIC) was used to evaluate model performance internally in the training dataset. The model with the smallest AIC is the preferred model. Goodness of fit in the validation dataset was assessed by estimating the deviance difference of the respective model when refitted on the validation dataset [15].

To evaluate model discrimination, we calculated PI = $\sum \widehat{\beta }x$, where *β* is the estimated parameter vector from the training dataset, in both the training data and the validation data. Using the 16th, 50th, and 84th percentile cutoffs from the training dataset, we stratified cumulative incidence plots in four different risk groups [16].

The Brier score measures the average squared difference between the observed and the estimated predictive outcome at a certain point in time. When the observed and predicted probabilities are close, the difference will be small (see Additional file 1 for details). The C-index was calculated from time-to-event data for a common length of follow-up. Calibration plots were generated for survival up to 2 years (see Additional file 1 for details).

For descriptive purposes, a summary comorbidity score was derived using the optimal set of comorbidity variables. A model using these variables together with age, sex, and information on previous ICU admissions was estimated in the training data. The linear predictor estimated for each individual when this model was used to predict probability of survival at 360 days in the validation data was used as a summary comorbidity score.

ICU readmission occurs at a non-negligible rate and is expected to be associated with patient outcome [17–19]. The potential for readmission to the ICU acting as a competing risk in survival analyses was evaluated by plotting cumulative incidence in risk categories based on the comorbidity score.

To quantify the added predictive value of a new comorbidity measure, the population was stratified according to age and Charlson comorbidity index, and within these strata, patients were stratified in quintiles of a comorbidity score based on a model with the new comorbidity variables but without age and sex as predictors. The observed time to death in these subsets was plotted, and hazard ratios comparing the lowest to the highest stratum-specific quartiles were calculated.

Data management was done in SAS version 9.4, and all statistical analyses were performed using R version 3.1.3.

## Results

### Baseline characteristics

During the 7-year study period, there were 230,056 ICU admissions among 188,965 individuals with a median age of 64 years on admission (Table 1). The most common admission diagnoses were trauma, circulatory failure, or respiratory failure (Additional file 1: eFig. S7, Additional file 1: eTable S2). In 14% of admissions, the patient had had another ICU admission during the previous year. Seventy-nine percent of all admissions had at least some baseline comorbidity identified, but only 54% of the admissions had a CCI > 0. The five most common comorbidities were infectious diseases, hypertension, ischemic heart diseases, injuries, and cardiac arrhythmias.

**Table 1:** Characteristics of admissions to the ICU. Note that a patient can have multiple admissions to the ICU and different characteristics on different admissions, and there can be multiple comorbidity categories associated with an ICU admission

	N with complete information	CCI 0 (*N* = 105,108)	CCI 1 (*N* = 43,080)	CCI 2–3 (*N* = 51,814)	CCI > 3 (*N* = 30,057)	All (*N* = 230,059)
Age	230,059					
0–15 years		0% (0)	0% (0)	0% (0)	0% (0)	0% (0)
16–24 years		15% (16,187)	4% (1545)	2% (802)	0% (145)	8% (18,679)
25–44 years		25% (25,946)	10% (4331)	5% (2679)	5% (1433)	15% (34,389)
45–64 years		30% (31,232)	31% (13,258)	27% (13,846)	29% (8791)	29% (67,127)
65–84 years		27% (28,057)	49% (20,898)	58% (30,308)	59% (17,605)	42% (96,868)
> 84 years		4% (3686)	7% (3048)	8% (4179)	7% (2083)	6% (12,996)
Sex	230,056					
Male		56% (59,110)	59% (25,273)	59% (30,812)	62% (18,659)	58% (133,854)
ICU admission diagnosis	230,059					
Trauma		32% (33,978)	13% (5678)	10% (5046)	8% (2384)	20% (47,086)
Circulatory		13% (13,456)	19% (8266)	17% (9022)	17% (5185)	16% (35,929)
Respiratory		9% (9520)	15% (6462)	18% (9435)	17% (5013)	13% (30,430)
Digestive tract		6% (6141)	8% (3548)	10% (4976)	14% (4094)	8% (18,759)
Infection		4% (4063)	4% (1792)	7% (3468)	8% (2306)	5% (11,629)
Endocrine		3% (3671)	5% (2301)	4% (2222)	5% (1576)	4% (9770)
Mental disorder		6% (5837)	2% (931)	1% (416)	0% (148)	3% (7332)
Nervous system		3% (2818)	2% (943)	2% (924)	1% (430)	2% (5115)
Malignancy/Hematology		1% (654)	0% (169)	4% (2101)	4% (1342)	2% (4266)
Urogenital		1% (1132)	1% (519)	2% (1188)	4% (1067)	2% (3906)
Pregnancy		2% (1899)	0% (71)	0% (12)	0% (3)	1% (1985)
Other		15% (15,530)	18% (7781)	17% (8958)	16% (4927)	16% (37,196)
Missing		6% (6409)	11% (4619)	8% (4046)	5% (1582)	7% (16,656)
SAPS3 score	123,119					
< 40		36% (20,846)	18% (3897)	10% (2598)	4% (751)	23% (28,092)
40–49		25% (14,506)	25% (5325)	19% (5122)	14% (2486)	22% (27,439)
50–59		18% (10,397)	25% (5373)	26% (7048)	25% (4306)	22% (27,124)
≥ 60		20% (11,400)	32% (6905)	46% (12,512)	56% (9647)	33% (40,464)
No of ICU admissions previous year	230,059					
0		92% (96,263)	84% (36,373)	82% (42,649)	78% (23,558)	86% (198,843)
1		6% (6343)	12% (5011)	13% (6705)	16% (4680)	10% (22,739)
2		1% (1453)	2% (1022)	3% (1531)	4% (1197)	2% (5203)
3		0% (499)	1% (303)	1% (447)	1% (401)	1% (1650)
4–5		0% (369)	1% (220)	0% (237)	1% (176)	0% (1002)
> 5		0% (181)	0% (151)	0% (245)	0% (45)	0% (622)
Time since previous ICU stay	230,059					
0–7 days		2% (2270)	5% (2003)	5% (2805)	6% (1725)	4% (8803)
8–30 days		2% (1739)	4% (1583)	4% (2136)	5% (1428)	3% (6886)
31–90 days		2% (1603)	3% (1095)	3% (1614)	4% (1223)	2% (5535)
91–365 days		3% (3244)	5% (2030)	5% (2623)	7% (2130)	4% (10,027)
> 365 days		92% (96,252)	84% (36,369)	82% (42,636)	78% (23,551)	86% (198,808)
Total ICU length of stay previous year	230,059					
< 24 h		96% (100,410)	90% (38,833)	88% (45,364)	84% (25,365)	91% (209,972)
1–7d		3% (3640)	7% (3120)	9% (4479)	11% (3404)	6% (14,643)
8–30d		1% (857)	2% (975)	3% (1624)	4% (1079)	2% (4535)
31–365d		0% (201)	0% (152)	1% (347)	1% (209)	0% (909)
Number of comorbidity categories	230,059					
0		46% (48,478)	1% (330)	0% (2)	0% (0)	21% (48,810)
1		19% (20,161)	14% (5967)	6% (2954)	0% (135)	13% (29,217)
2		14% (14,823)	22% (9415)	10% (5401)	3% (1038)	13% (30,677)
3		9% (9612)	22% (9305)	16% (8471)	6% (1950)	13% (29,338)
4		6% (5962)	17% (7419)	19% (9623)	11% (3198)	11% (26,202)
5		3% (3153)	11% (4656)	17% (8674)	13% (3927)	9% (20,410)
6		2% (1609)	7% (2864)	13% (6581)	15% (4366)	7% (15,420)
7		1% (743)	4% (1587)	9% (4410)	14% (4279)	5% (11,019)
8		0% (336)	2% (854)	5% (2663)	12% (3684)	3% (7537)
9		0% (125)	1% (366)	3% (1521)	9% (2785)	2% (4797)
> 9		0% (106)	1% (317)	3% (1514)	16% (4695)	3% (6632)
Comorbidity categories	230,059					
Infectious disease		13% (13,247)	31% (13,316)	44% (22,762)	57% (17,231)	29% (66,556)
Hypertension		9% (9737)	36% (15,654)	45% (23,107)	54% (16,090)	28% (64,588)
Ischemic heart disease		5% (5244)	24% (10,451)	34% (17,529)	42% (12,741)	20% (45,965)
Injury		14% (15,130)	20% (8634)	20% (10,489)	23% (6935)	18% (41,188)
Cardiac arrhythmias		6% (6740)	22% (9395)	28% (14,606)	32% (9684)	18% (40,425)
Neurological disease		9% (9603)	19% (8181)	23% (11,941)	28% (8445)	17% (38,170)
Diabetes		0% (37)	16% (6997)	26% (13,633)	46% (13,767)	15% (34,434)
Chronic pulmonary disease		1% (1536)	19% (8126)	25% (13,203)	29% (8789)	14% (31,654)
Bone/muscle disease		7% (7723)	15% (6651)	18% (9508)	23% (7033)	13% (30,915)
Tumor non-metastatic		0% (109)	0% (104)	27% (13,876)	40% (11,919)	11% (26,008)
Peripheral vascular disease		1% (1482)	13% (5734)	16% (8441)	31% (9326)	11% (24,983)
Cerebrovascular disease		0% (16)	15% (6476)	17% (8626)	24% (7071)	10% (22,189)
Alcohol abuse		9% (9704)	10% (4487)	7% (3568)	12% (3650)	9% (21,409)
Renal disease		2% (1948)	5% (2026)	13% (6537)	32% (9756)	9% (20,267)
Other anemias		2% (2602)	7% (2866)	13% (6480)	24% (7139)	8% (19,087)
Valvular disease		5% (4900)	11% (4639)	10% (5195)	9% (2825)	8% (17,559)
Depression		8% (8577)	7% (2870)	6% (2930)	6% (1823)	7% (16,200)
Poisoning		9% (9097)	7% (2893)	4% (2128)	5% (1431)	7% (15,549)
Drug abuse		6% (6715)	9% (3757)	6% (2962)	6% (1913)	7% (15,347)
Fluid balance disorder		2% (2406)	6% (2436)	8% (4028)	14% (4068)	6% (12,938)
Other endocrine disease		2% (2371)	7% (2807)	8% (4282)	11% (3208)	6% (12,668)
Hepatic disease		0% (372)	7% (3218)	6% (3092)	18% (5321)	5% (12,003)
Rheumatic/autoimmune disease		0% (513)	4% (1767)	7% (3490)	8% (2504)	4% (8274)
Pulmonary circulation disorders		1% (1056)	3% (1246)	5% (2449)	6% (1711)	3% (6462)
Tumor metastatic		0% (3)	0% (0)	0% (19)	20% (6156)	3% (6178)
Obesity		1% (1500)	3% (1196)	3% (1684)	5% (1397)	3% (5777)
Hematological disease		1% (695)	2% (707)	4% (2029)	7% (1974)	2% (5405)
Blood loss anemia		1% (695)	2% (753)	3% (1548)	7% (1971)	2% (4967)
Psychoses		2% (2441)	2% (1059)	2% (923)	2% (492)	2% (4915)
Hematological malignancy		0% (11)	0% (9)	5% (2736)	5% (1438)	2% (4194)
Transplantation-related disorder		0% (65)	0% (181)	2% (897)	4% (1252)	1% (2395)
Coagulopathy		0% (367)	1% (381)	1% (678)	2% (650)	1% (2076)
Malnutrition		0% (361)	1% (344)	1% (649)	2% (501)	1% (1855)
Immunodeficiency		0% (107)	0% (139)	0% (242)	1% (394)	0% (882)

### Development of a prediction model for the impact of baseline comorbidity on time to death

**Fig. 1:**
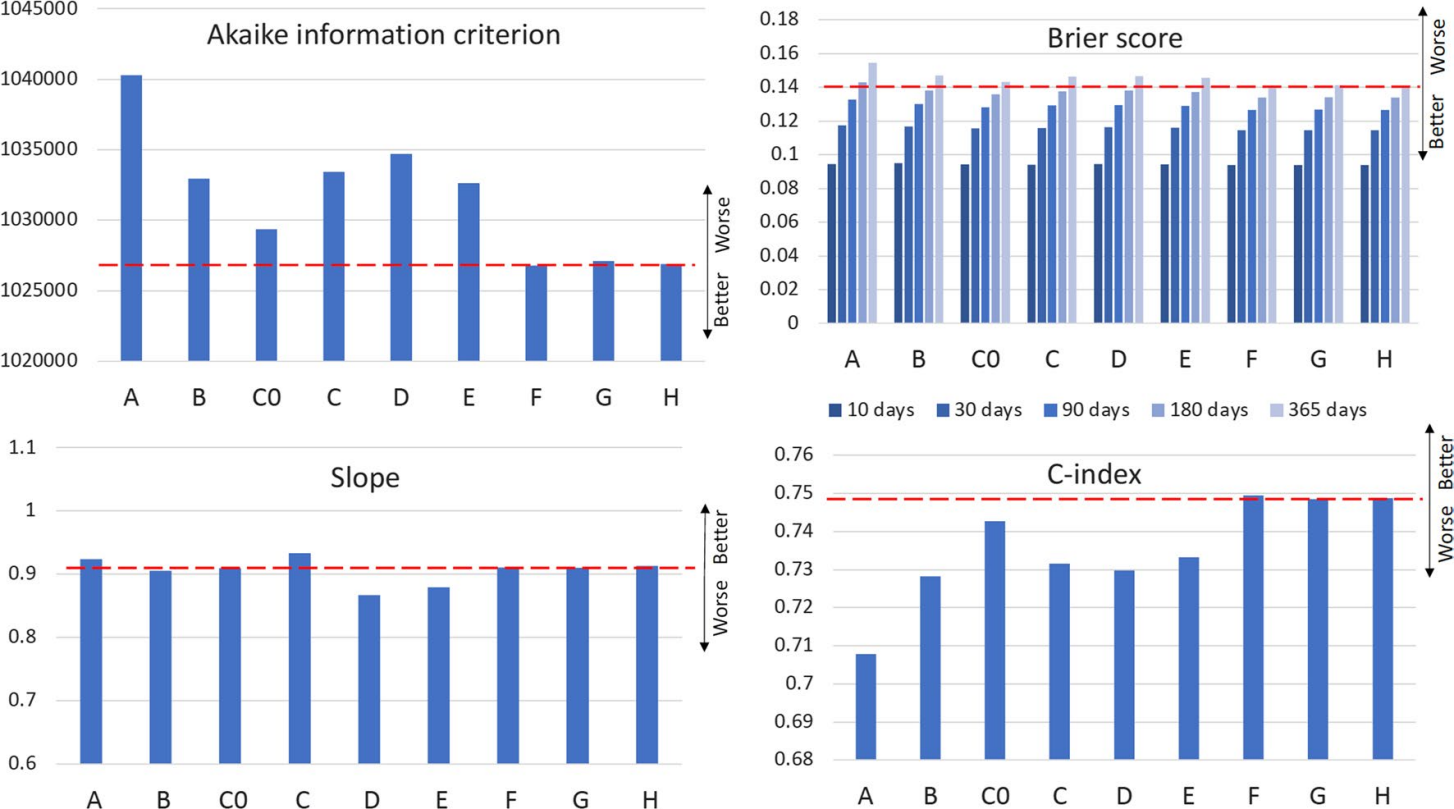
Measures comparing predictive ability of different multivariable models evaluated. AIC = Akaike information criterion. Model A: Age + sex + [*Variables indicating number of intensive care unit (ICU) admissions, total length of stay, and time since last ICU discharge during 365 days prior to the index admission date*]. Model B: Model A + Charlson comorbidity index. Model C0: Model A + [*Variables indicating the presence of at least one admission with a principal diagnosis in the respective comorbidity category*]. Model C: Model A + [*Variables indicating the number of admissions with a principal diagnosis in the respective comorbidity category*]. Model D: Model A + [*Variables indicating the number of admissions with a principal or secondary diagnosis in the respective comorbidity category*]. Model E: Model C + [*Variables indicating the sum of length of hospital stays with a main diagnosis in the respective comorbidity category*]. Model F: Model C + [*Variables indicating the interval in days since discharge from the most recent hospital stay with a main diagnosis in the respective comorbidity category*]. Model G: Model A + [*Variables indicating the interval in days since discharge from the most recent hospital stay with a main diagnosis in the respective comorbidity category*] Model H: Model A + [*Variables indicating the interval in days since discharge from the most recent hospital stay with a main diagnosis in the respective comorbidity category*] + [*Variables indicating the sum of length of hospital stays with a main diagnosis in the respective comorbidity category*]

Quantitative information on comorbidity, extracted from hospital discharge information in 36 comorbidity categories, was compared to a basic model with age, sex, and ICU admission history. It was also contextualized against CCI as the conventional comorbidity measure (Additional file 1: eTable S8). Best overall predictive performance was seen when variables indicating the time interval from the most recent hospital stay were added for each comorbidity category (models F–H in Fig. 1). The count of hospital admissions or the total hospital length of stay in the comorbidity categories did not appear to contribute any further to predictive ability.

The model that included variables indicating the time since last hospital stay and the total length of hospital stay for each comorbidity category (model H) was selected as the final model to use for further description of comorbidity model performance. The final comorbidity variables used were defined solely based on principal diagnoses from previous hospital admissions. Secondary diagnoses did not add substantially to predictive ability.

### Evaluation of comorbidity variables in a validation dataset

To reduce the problem of overly optimistic estimation of model performance, we also evaluated goodness of fit of the different models derived in the training dataset in a validation dataset. The deviance difference as a fraction of χ^2^_0.95_ was lowest for model F (2.2), G (2.4), and H (2.3), indicating best goodness of fit (Additional file 1: eTable S3).

The prediction models derived from the training set were then used to stratify patients in both the training and validation datasets according to their predicted mortality rate at baseline. The observed mortality in these strata of the validation dataset and the training dataset was very similar, indicating similar performance of the prediction model in the training and validation datasets (Additional file 1: eFig. S8). The increased separation of survival curves between strata illustrates the increased discriminative ability of models with more extensive information on comorbidity.

The smallest/best Brier scores were seen for model F (0.141), G (0.141), and H (0.141) (Additional file 1: eTable S4). The difference in Brier score between the basic model A and the more extensive comorbidity models was larger for longer-term than short-term outcomes, with lower Brier scores in the more extensive models.

Best discrimination (highest C-index values) was seen for model F (0.749), G (0.748), and H (0.749) (Additional file 1: eTable S5). The calibration slope closest to the optimal value of 1 was seen for model C, while models D and E deviated most from a slope of 1 (Additional file 1: eTable S6). For model H, there is a discrepancy between predicted and observed morality risk mainly in the subset with high mortality (Additional file 1: eFig. S10). The deviation is most apparent for short-term mortality. Looking at the calibration plots for the simple models, it is evident that the apparent good calibration is related to the scarcity of data for these models in the high-risk part of the risk range (Additional file 1: eFig. S9).

### Added value for prediction of mortality compared to the CCI

To illustrate the added value of a prediction model that includes time since last hospital stay and total length of hospital stay within comorbidity categories (model H), we used this model to calculate a summary comorbidity score and compared this to the CCI (Additional file 1: eTable S8).

**Fig. 2:**
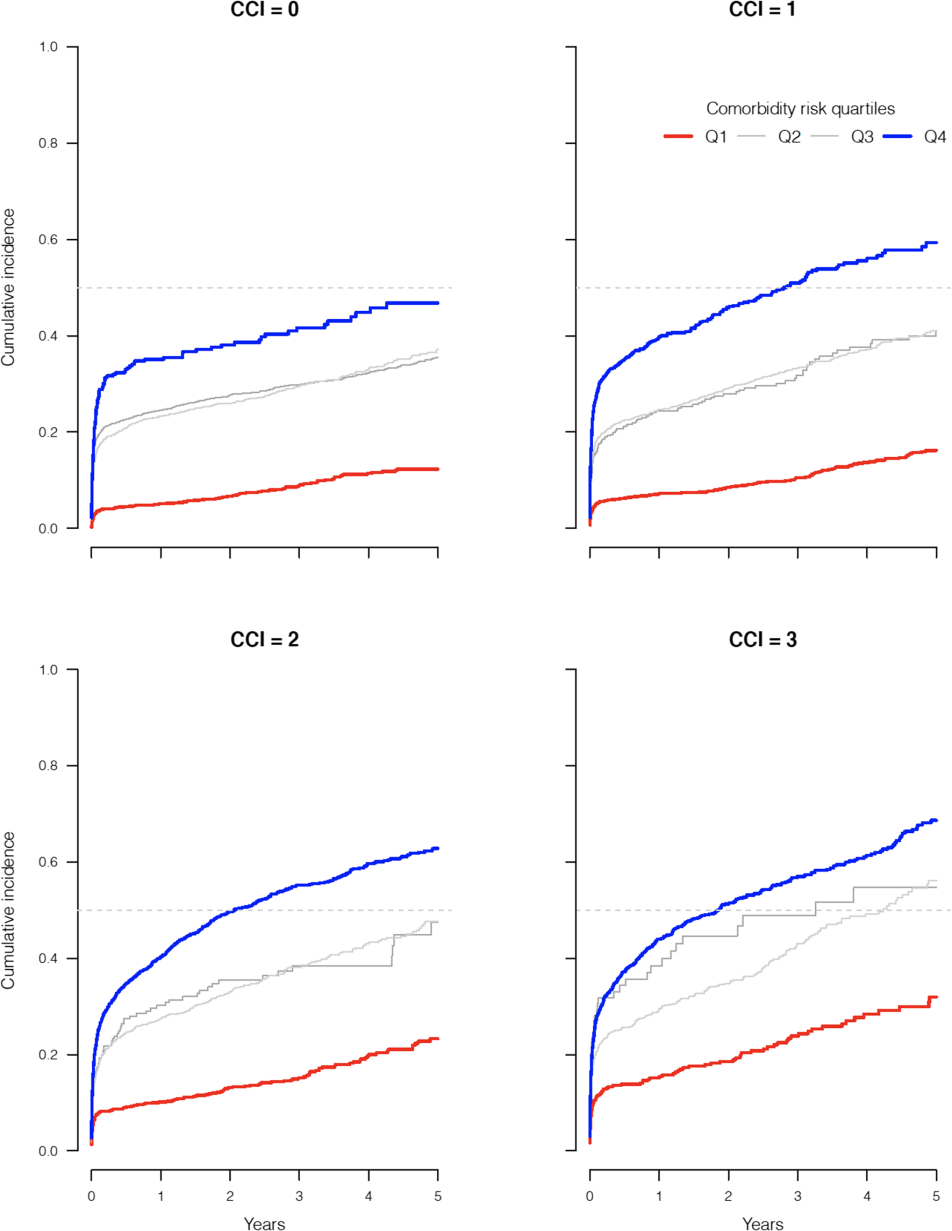
Survival of patients in the age group 71–75 years having a Charlson comorbidity index 1–4. The survival probability is displayed stratified by quartiles of predicted probability of survival as measured by the linear predictor from a model with optimal selection of comorbidity variables (model H; see Additional file 1: eTable S8 for description) but without age and sex as predictors in the model

**Fig. 3:**
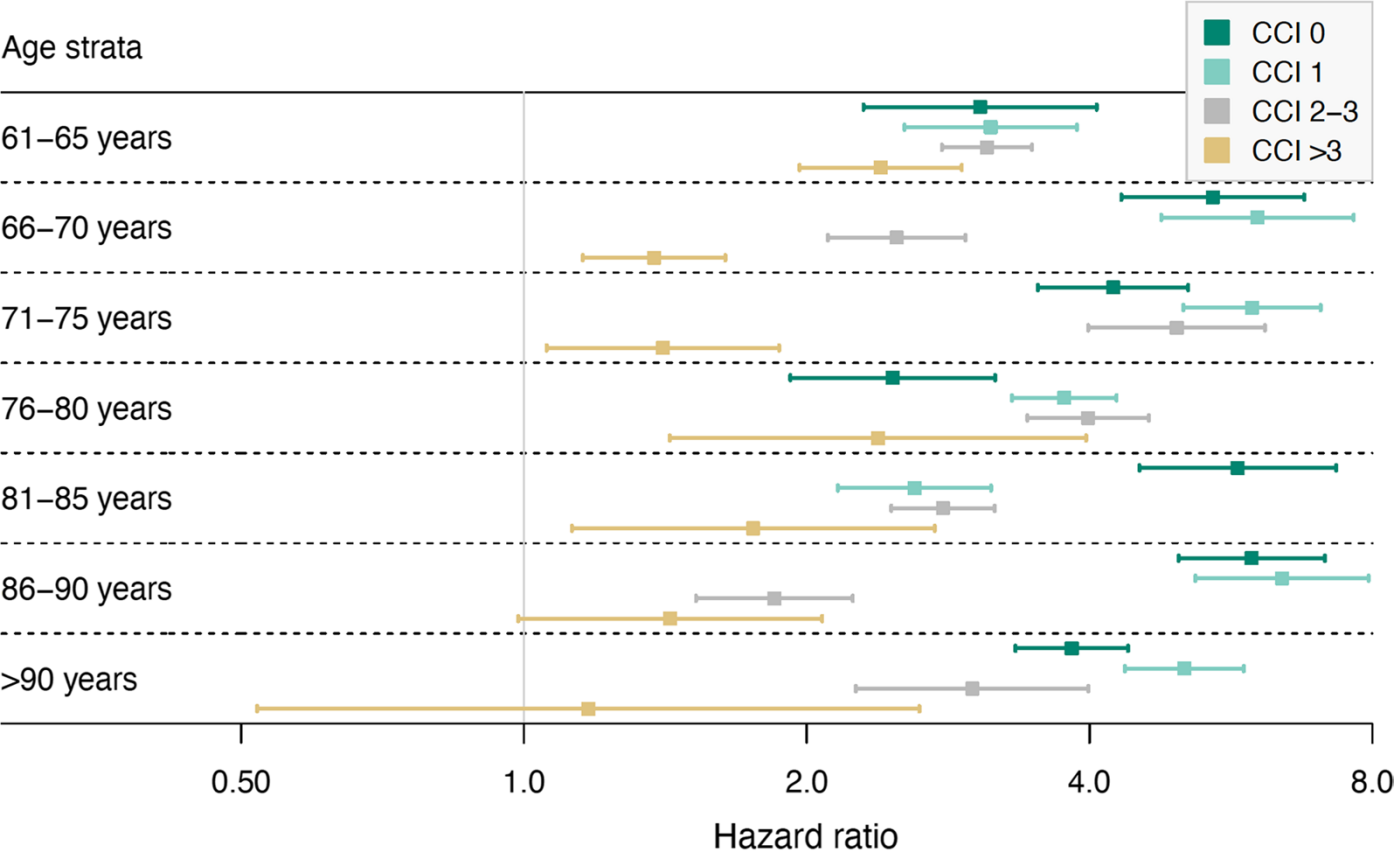
Hazard ratios within strata of age and the Charlson comorbidity index (CCI), comparing the survival probability in the lowest to the highest quartiles of our proposed optimized summary comorbidity measure. The linear predictors from model H (see Additional file 1: eTable S8 for description), but excluding age and sex from the model, were used as a summary measure of comorbidity for each individual

Even within restricted age strata with a specific CCI, this summary score based on model H still provided further clear separation of risk categories, also within the subgroup with CCI = 0 (Fig. 2). As an example, in the subset of 4208 patients aged 61–65 years with CCI = 2 one-year survival was 91.3% (95% CI 89.4% to 93.2%) in the lowest quartile, and 66.9% (95% CI 64.7% to 69.2%) in the highest quartile. This ability to separate baseline risk was consistent across age and CCI strata but less pronounced when CCI was high (Fig. 3).

### Added value for prediction of mortality compared to the SAPS

**Fig. 4:**
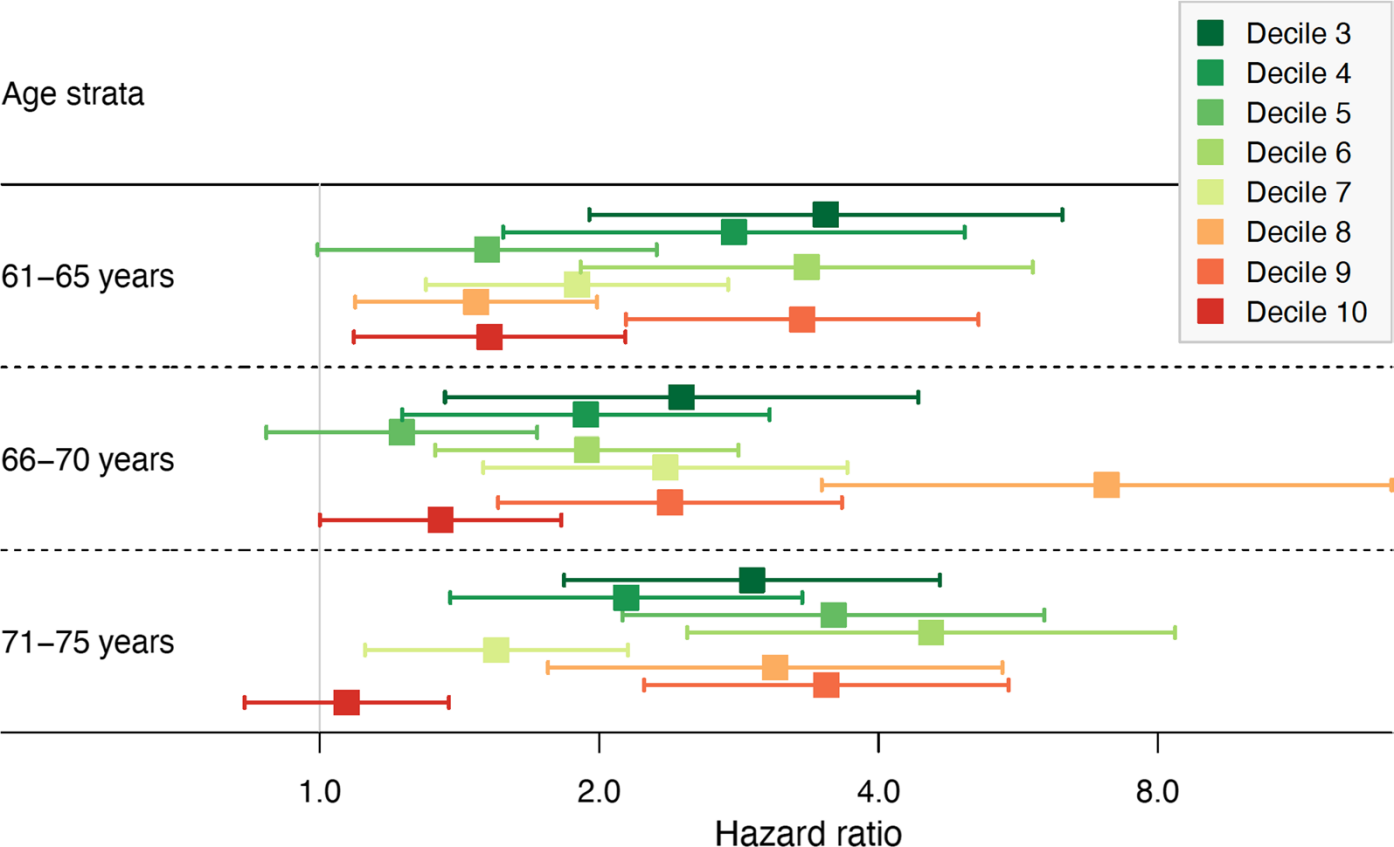
Hazard ratios within strata of age and the Simplified Acute Physiology Score (SAPS), comparing the survival probability in the lowest to the highest quartiles of our proposed optimized summary comorbidity measure. The linear predictors from model H (see Additional file 1: eTable S8 for description), but excluding age and sex from the model, were used as a summary measure of comorbidity for each individual. Hazard ratios could not be estimated for the lowest SAPS strata

The optimal comorbidity model (model H) also provided separation of predicted mortality within risk strata defined by the level of the baseline SAPS score (Additional file 1: eFig. S12). This was mainly seen in the medium SAPS range, but not as evident in the SAPS strata with low or high baseline mortality risk (Fig. 4). As an example, in the subset of 1712 patients aged 61–65 years within the fifth SAPS decile, one-year survival was 88.8% (95% CI 84.4% to 93.4%) in the lowest quartile and 71.7% (95% CI 67.1% to 76.7%) in the highest quartile.

### Readmission to the ICU as a potential competing risk

The training dataset consisted of 153,341 admissions with 46,284 deaths and 30,515 readmissions during a median follow-up of 2.5 years (range 0–7 years). In the validation dataset with 76,715 admissions, there were 15,941 deaths and 10,579 readmissions during a median follow-up of 0.6 years (range 0–2 years) (Additional file 1: eFig. S3-5). Readmission was more common in younger patients (Additional file 1: eFig. S6).

When the study population was stratified based on the summary comorbidity score calculated with model H, the observed mortality was clearly related to the predicted risk (Additional file 1: eFig. S13). No such clear relationship could, however, be seen for the cumulative risk for readmission to the ICU. Readmission to the ICU was therefore not considered as an important competing risk for mortality. Censoring of follow-up at the time of readmission to the ICU was consequently assumed to be non-informative.

### Comorbidity variables with little impact on predictive ability

Variables merely indicating the presence of some previous admission with a main diagnosis in the respective comorbidity category did not notably improve mortality prediction compared to a model including the CCI (Fig. 1, Additional file 1: eTable S8). No improvement was seen if variables indicating the number of such admissions in each category were used instead. Adding also the number of previous admissions with a secondary diagnosis from each category (model D) or variables indicating the total length of stay with a main diagnosis for the respective comorbidity category (model E) did not improve model performance notably.

### Subgroup analysis in patients with multiple comorbidities

The 28,854 ICU admissions in the validation dataset subgroup with comorbidity from at least two different categories were older, had somewhat higher SAPS score at baseline, and higher Charlson comorbidity index (Additional file 1: eTable S7). The mortality predictions based on the variables in model H were less discriminative in this subgroup (C-index = 0.72) compared to the overall validation dataset (C-index = 0.75), but calibration was largely comparable (Additional file 1: eFig. S10). The improvement of the extensive model H compared to most basic model A was more apparent in the subgroup (Additional file 1: eFig. S8).

In the subgroup analyses, the extensive comorbidity models in general had higher Brier scores compared to the overall validation dataset, but the differences between the basic model and the more extensive models were larger, especially for long-term follow-up (Additional file 1: eTable S4).

A prediction model based on age, sex, and SAPS score was developed in the training dataset and applied to the validation dataset (Additional file 1: eFig. S12). Its performance in the subgroup with multiple comorbidities was not as good as in the overall validation dataset. The Brier score at 365 days increased from 0.13 to 0.16 when restricted to this subgroup. Somewhat unexpectedly the same phenomenon was observed also for short-term mortality, with an increase from 0.11 to 0.14 at 90 days, and 0.08 to 0.10 at 10 days of follow-up (Additional file 1: eFig. S11, Additional file 1: eTable S9).

## Discussion

### Main results summary

Prediction of long-term all-cause mortality in a general ICU population was most substantially improved by including the time interval from the most recent hospital stays for each of 36 comorbidity categories. Time since the last hospital stay and total length of stay for each comorbidity category provided further separation of risk categories also within strata of age, the Charlson comorbidity index, and within intermediate SAPS3 risk strata. In the subgroup with multiple comorbidities, the optimal comorbidity measure was less discriminative, but calibration was better compared to that seen in the full validation dataset.

### The potential problem with readmissions

ICU readmission occurs at a non-negligible rate and is expected to be associated with patient outcome [17–19]. It therefore constitutes a potential competing event that bias studies of long-term mortality. In this study, we did not find a notable association between severity of comorbidity and the risk for readmission to the ICU. Readmission to the ICU was therefore not considered further as a competing risk for mortality in our analyses.

### Failed attempts to increase granularity of information

Only some of the information extracted from the history of hospital discharge diagnoses proved clearly useful. Secondary diagnoses did not contribute to predictive ability beyond the principal diagnoses. The count of hospital admissions within a comorbidity category did not improve mortality prediction. The length of hospital stays added only marginally to predictive ability.

### Importance of effective comorbidity measures based on register data

Summarizing ICU patients’ histories of hospital discharge diagnoses has several advantages as a measure of comorbidity. No additional manual registration is required by the ICU staff, and comparisons of ICU patients to non-ICU populations can be adequately adjusted for comorbidity. Combination with other readily available health-care data could potentially further facilitate baseline risk stratification. We have previously demonstrated the utility of a panel of baseline laboratory data routinely available in electronic health-care records, for mortality risk stratification of ICU patients [20].

### Clinical relevance of the improved model performance

When models including the time interval from the most recent hospital stay with the respective comorbidity category and length of stay were used to stratify the study population, these strata were found to be well separated also within strata of Charlson comorbidity index even when this index suggested that no comorbidity was present. Also, within intermediate strata of the SAPS score the comorbidity variables we derived provided further separation of predicted mortality risk. The failure to provide further separation in SAPS strata with high baseline mortality risk may be explained by a high mortality risk associated with the admission diagnosis and consequently relatively less impact of comorbidity [21].

### Strengths and limitations of our study

A strength of our study is the long-term follow-up with a comprehensive and population-based data collection with almost complete coverage of all Swedish ICUs. Differences in coding practices and health-care system characteristics between countries may, however, limit generalizability and hamper international comparisons [22, 23].

Also, statistical measures such as the C-index are difficult to interpret in terms of actual added value of new predictors. To provide a more practical illustration of the added value of the new comorbidity measures, we demonstrated the ability to provide risk separation within strata of age, the Charlson comorbidity index, and the SAPS score.

Importantly, our study is focused on the assessment of comorbidity from the patient’s history of health-care utilization. While not within the scope of our study, it is evident that the immediate reason for ICU admission, its severity, as well as other patient characteristics such as socioeconomic status [24–26] are key predictors that need separate attention. This scope of this study was to optimize measurement of comorbidity and did not aim to develop an overall optimized long-term mortality model.

In conclusion, measures of baseline comorbidity can be improved by adding variables indicating the time interval from the most recent hospital stay with the respective comorbidity category. Using such a more comprehensive prediction model provided separation of risk categories also within strata of age, the Charlson comorbidity index, and intermediate SAPS3 strata.

## Take-home message

Measures of baseline comorbidity can be improved by adding variables indicating the time interval from the most recent hospital stay with the respective comorbidity category. Using such a more comprehensive prediction model provided separation of risk categories also within strata of age, the Charlson comorbidity index and intermediate SAPS3 strata.

## Supplementary Information

**Additional file 1.** Optimized diagnosis-based comorbidity measures for all-cause mortality prediction in a national population-based ICU population - Supplementary online only material.

## Data Availability

Data are available from the corresponding author on reasonable request pending appropriate permissions and data access agreements.
