# Supplementary material for: Optimized diagnosis-based comorbidity measures for all-cause mortality prediction in a national population-based ICU population
Source: Crit Care. 2022 Oct 6;26:306. doi: 10.1186/s13054-022-04172-0 (PMC9535950; doi:10.1186/s13054-022-04172-0)

# Optimized diagnosis-based comorbidity measures for all-cause mortality prediction in a national population-based ICU population

# - Supplementary online only material

## Definition of comorbidity categories

**eTable S1.** Operational definitions of comorbidity categories using ICD-9 or ICD-10. The definitions according to Elixhauser et al have been expanded and new categories added [6].

|  | Comorbidity category | ICD-9 | ICD-10 |
| --- | --- | --- | --- |
| 1 | Congestive heart failure (CHF) | 398, 393A, 402A, 402X, 404A, 404B, 404X, 425, 428, 429 | I13, I42, I50, I51 |
| 2 | Ischemic heart disease (IHD) | 410-414 | I20-I25 |
| 3 | Valvular disease (Valv) | 394-397, 421, 424 | I05-I08, I091, I33-I39 |
| 4 | Cardiac arrythmias (Ary) | 427, 427 | I44-I49, R00 |
| 5 | Pulmonary circulation disorders (Pulmcirc) | 415-417 | I26-I28 |
| 6 | Hypertension (HT) | 401-405 | I12, I15, I109, I119 |
| 7 | Cerebrovascular disease (CVD) | 430-438 | I60-I69 |
| 8 | Peripheral vascular disease (Perivasc) | 440-447, 452, 453, 456 | I70-I82, I85-I89, R029 |
| 9 | Neurologic disease (Neurol) | 290, 293, 294, 310, 320-359, 780A, 781C, 781D, 784D, 784F | R40, R41, R47, R48, G00-G99, F00-F09, R25-R29, R296, R298 |
| 10 | Chronic pulmonary disease (CPD) | 490-505, 506E, 506X, 508B, 515-517, 519B, 519E, 519W | D860, D862, E662, E840, J40-J67, J684, J701, J703, J84, J850, J953, J961, J969, J98, J99 |
| 11 | Infectious disease (Inf) | 001-066, 070B, 071-078, 079A-079D, 079W, 079X, 080-139, 460-466, 473, 480-491, 510, 511, 590, 595, 597, 790H, 790W, 998F, 999D, | A00-B09, B25-B99, B159, J00-J32, J851-J869, M00-M01, M726, N300, N390, T880 |
| 12 | Diabetes (Diab) | 250 | E10-E14 |
| 13 | Other endocrine disorders (Xendo) | 251-259 | E00-E07, E15-E35, E89 |
| 14 | Renal disease (Renal) | 403, 404, 580-589 | N00-N12, N14-N19, V42A, V45B, V56, R34, R392, Z992 |
| 15 | Hepatic disease (Hepatic) | 070A, 070C-070X, 456A, 456B, 570-573 | B150, B16-B18, I85, K70-K77, V42H |
| 16 | Immune deficiencies incl. HIV (Immundef) | 173, 279, 079J | D89, B20-B24, D80-D84, V02J |
| 17 | Hematological malignancies (Hemomal) | 200-208, | C81-C96, D477-D479 |
| 18 | Other hematological disease (Hemodis) | 288, 289 | D45, D46, D471-D473, D69-D77 |
| 19 | Solid tumour without metastases (tumournomet) | 140-195 | C00-C76, Z510, Z511, Z926 |
| 20 | Metastatic cancer (Tumourmet) | 196-199 | C77-C80, C979 |
| 21 | Rheumatic disease (Collagen) | 279N, 710, 713D, 713H, 714, 720, 725, 729A-729E, | M05-M09, M30-M35 |
| 22 | Coagulopathy (Coag) | 286, 287 | D65-D68 |
| 23 | Obesity (Obese) | 278 | E65, E66 |
| 24 | Nutritional deficiences (Nutr) | 260-269 | E40-E64, E90 |
| 25 | Fluid and electrolyte disorders (Fluid) | 276 | E86, E87, R631 |
| 26 | Blood loss and anemia (Bleed) | 285B | D629 |
| 27 | Deficiency and other anemia (Anemia) | 280-284, 285A, 285W, 285X | D50-D61, D64 |
| 28 | Alcohol abuse (Alco) | 291, 303, 305A, 790D | F10, K70, K852, R780, Y90, Y91, Z502, Z721 |
| 29 | Drug abuse (Drug) | 292, 304, 305B, 305X | F11-F19, R781-R786, Z503, Z722, |
| 30 | Psychoses (Psycho) | 295, 297-299, 780B | F20-F29, R44 |
| 31 | Affective disorders (Affect) | 296, 311 | F30-F39 |
| 32 | Other psychiatric disorders (Xpsych) | 300-302, 307-309, 312-319 | F40-F99, Z504 |
| 33 | Transplantation (Tx) | 996W | V42, T86, Z940-Z944 |
| 34 | Bone or muscle disease (Bonemusc) | 715-738 | M12-M25, M40-M83, M858, M859, M86-M90, M99 |
| 35 | Injury (Injury) | 800-959, 990-994, 995F | S00-T35, T66-T79, T90-T98 |
| 36 | Poisoning (Intox) | 960-989 | T36-T65 |

## Functional form of continuous variables

Visual inspection of generalized additive model (GAM) plots for the outcome death in the training data set was used to verify that age and length of stay of previous ICU admissions could be modeled as linear effects

**eFigure S1.** Generalized Additive Model (GAM) plot of age versus the outcome in the training data set in presence of the covariates sex, time since most recent previous ICU stay (strata), number of previous ICU admissions (categorical) and total length of previous ICU stays (numerical). Blue dots correspond to observed values, but a single dot can represent many observations. With reference to this figure, we model age for the primary outcome death with a linear term.


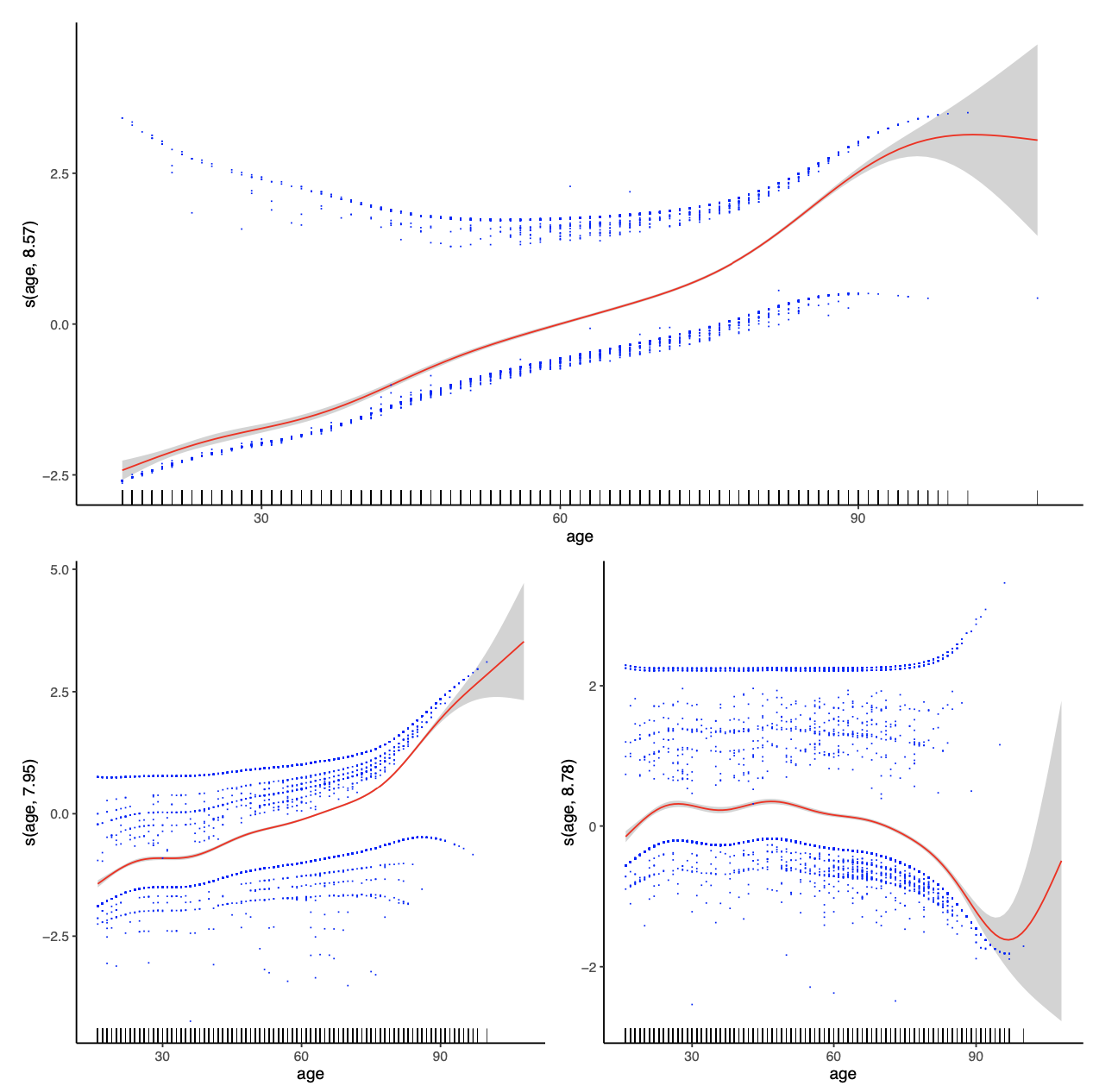


**eFigure S2:** Generalized Additive Model (GAM) plot of the variable indicating total length of stay (hours) of previous ICU admissions versus the outcome in the training data set in presence of the covariates sex, time since most recent previous ICU stay (strata), and number of previous ICU admissions (categorical). Blue dots correspond to observed values but a single dot can represent many observations. With reference to this figure we model total length of stay of previous ICU admissions for the primary outcome death with a linear term.


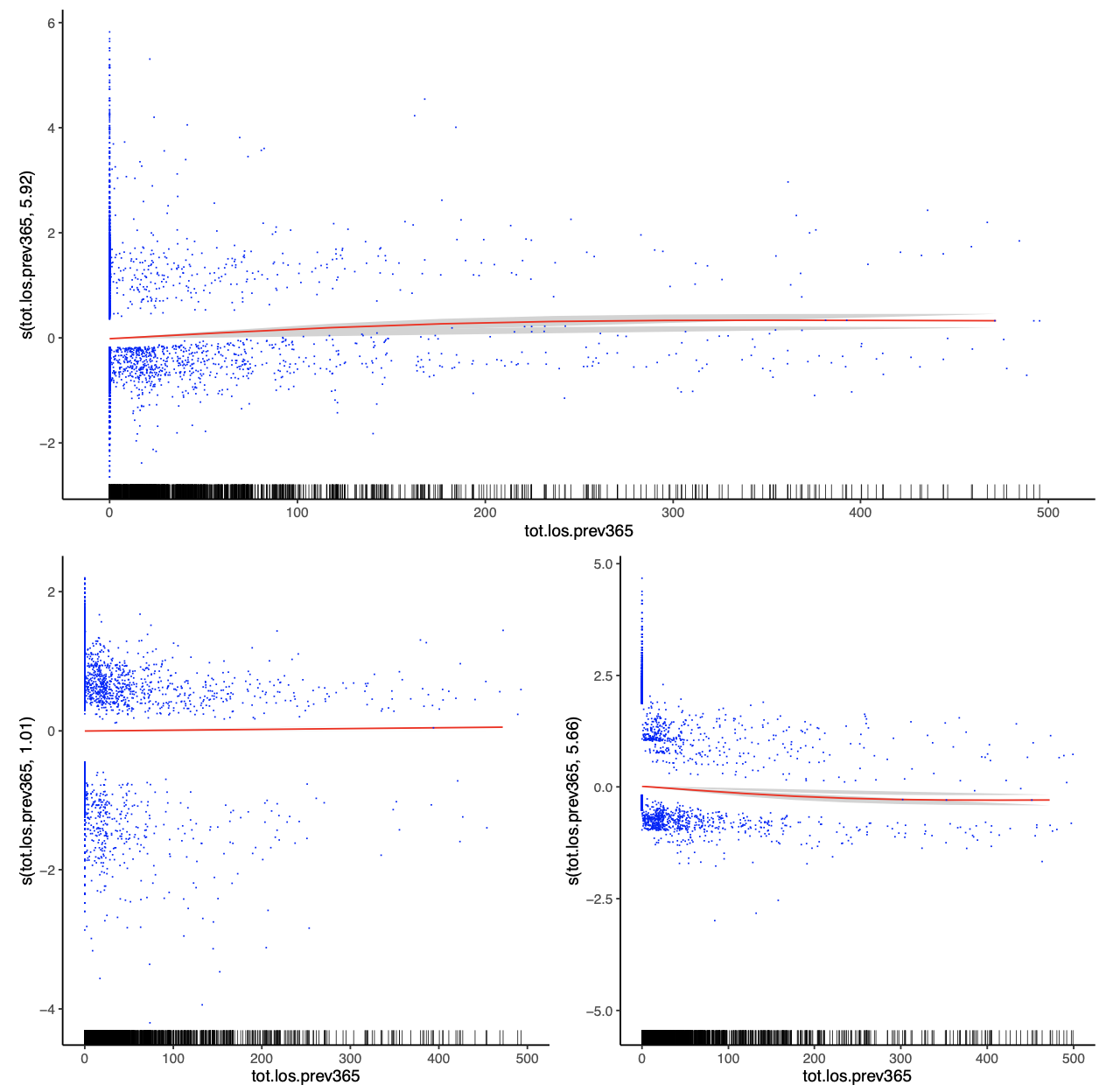


**eFigure S3**. Cumulative incidence of the primary outcome (death) estimated by the method of Kaplan Meier in the training data set (to the left) and the in the validation data set (right). The vertical dashed line in the figure to the left corresponds to the maximum follow-up in the validation data set. Note that in the graph of the validation data set the comorbVal is a subset of the validation data consisting of patients with at least two comorbidities.

**
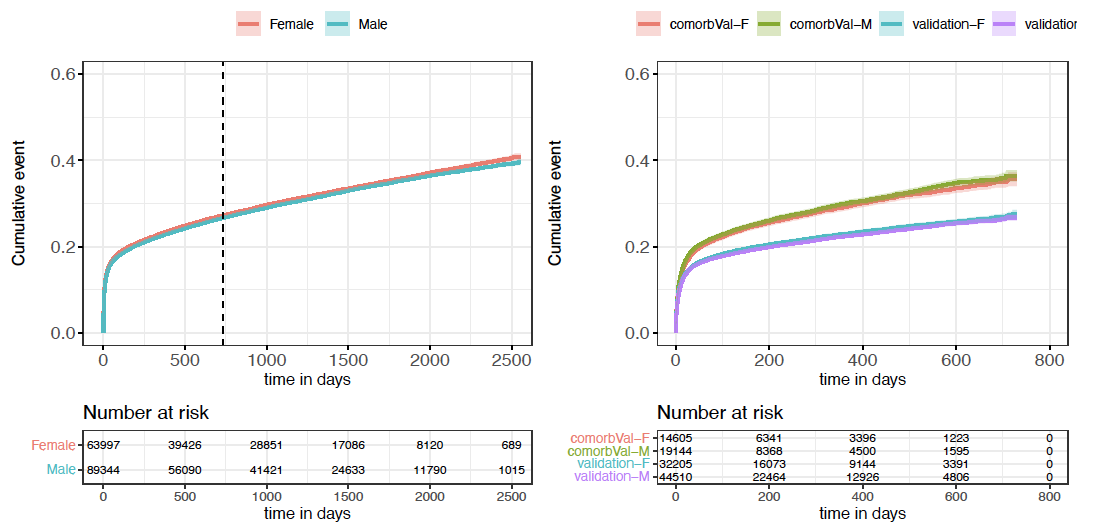
**

**eFigure S4.** Cumulative incidence of the secondary outcome readmission estimated by the method of Kaplan Meier in the training data set (to the left) and the in the validation data set (right). The vertical dashed line in the figure to the left corresponds to the maximum follow-up in the validation data set. Note that in the graph of the validation data set the comorbVal is a subset of the validation data consisting of patients with at least two comorbidities.

**
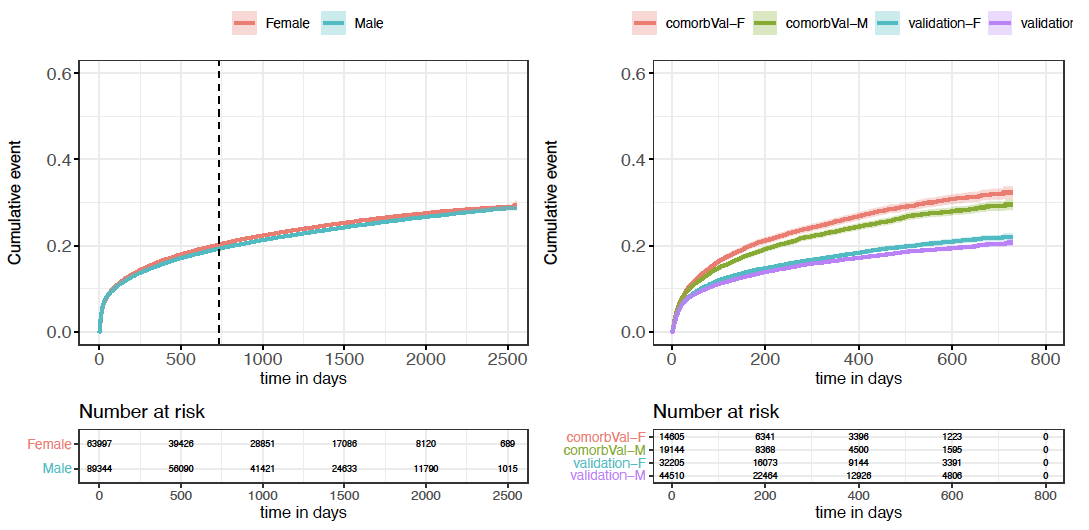
**

**eFigure S5.** Cumulative incidence of the secondary outcome composite (death or readmission) estimated by the method of Kaplan Meier in the training data set (to the left) and the in the validation data set (right). The vertical dashed line in the figure to the left corresponds to the maximum follow-up in the validation data set. Note that in the graph of the validation data set the comorbVal is a subset of the validation data consisting of patients with at least two comorbidities.

**
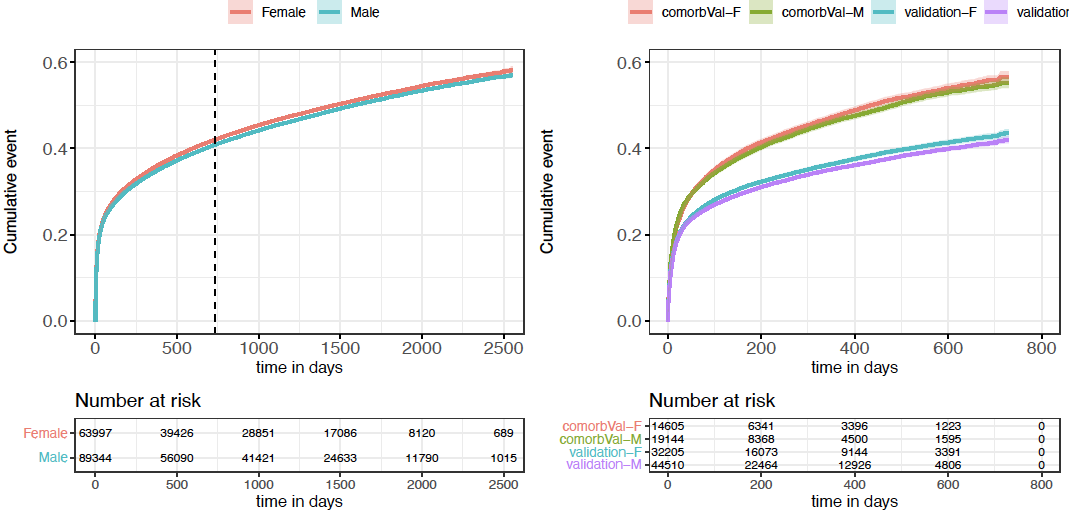
**

**eFigure S6.** Age density stratified on the outcomes death, readmission and the composite of these two.

**
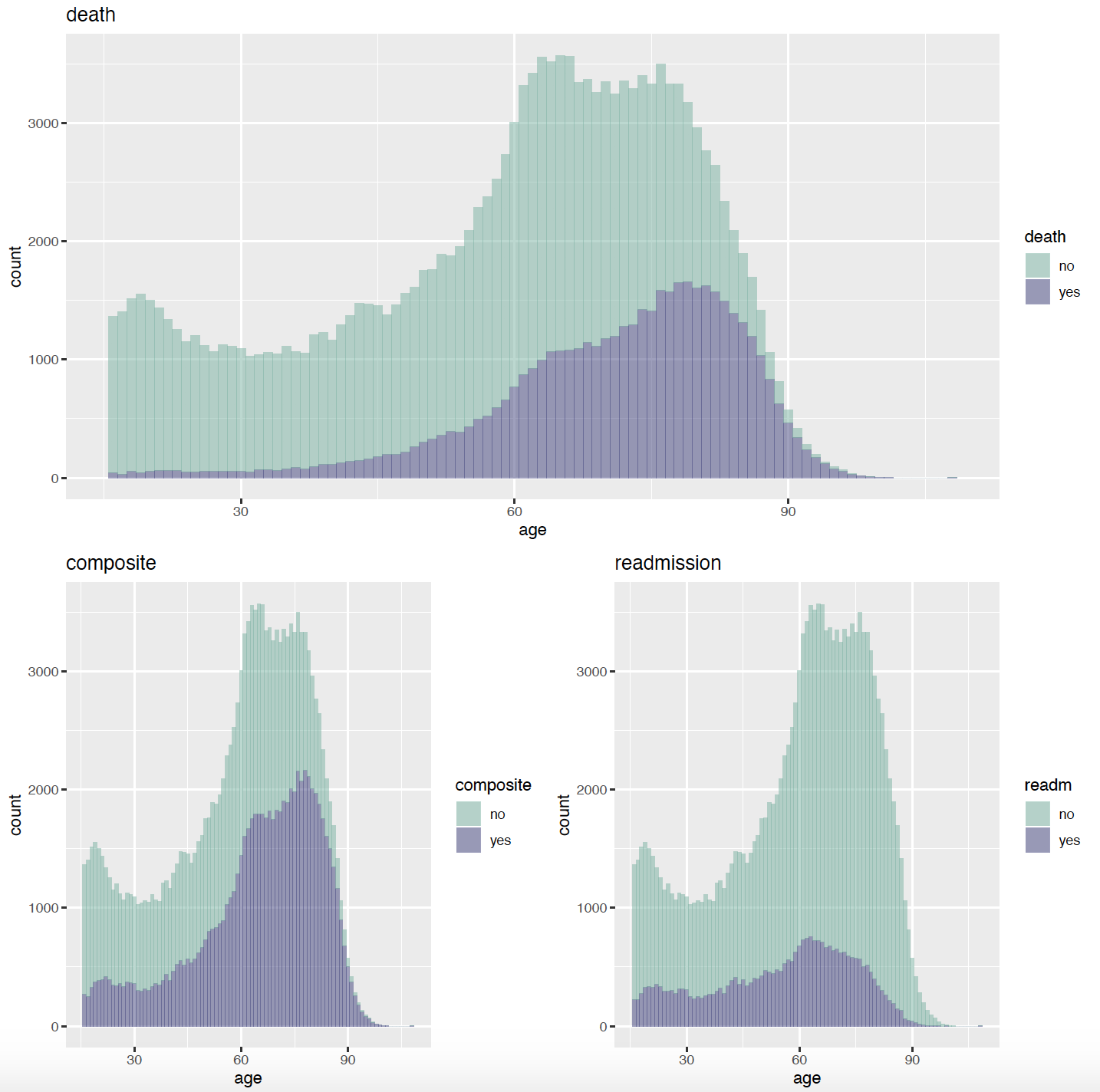
**

**eFigure S7.** Distribution of the number of hospital admissions within each comorbidity category during the 5-year look-back period prior to the index date for ICU admission. The data is presented for the training and validation dataset and when the number of admissions is greater than 0 but less than 11.


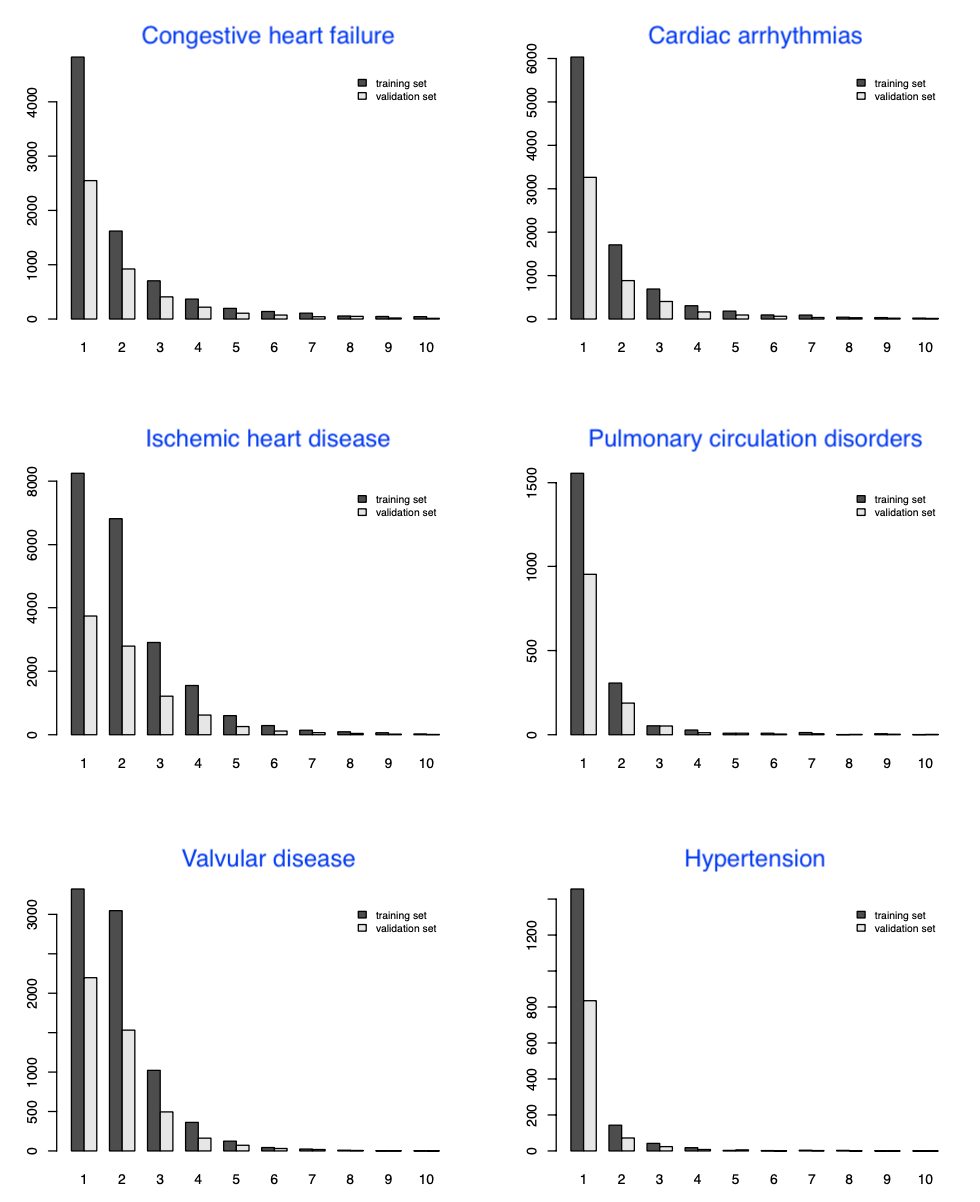


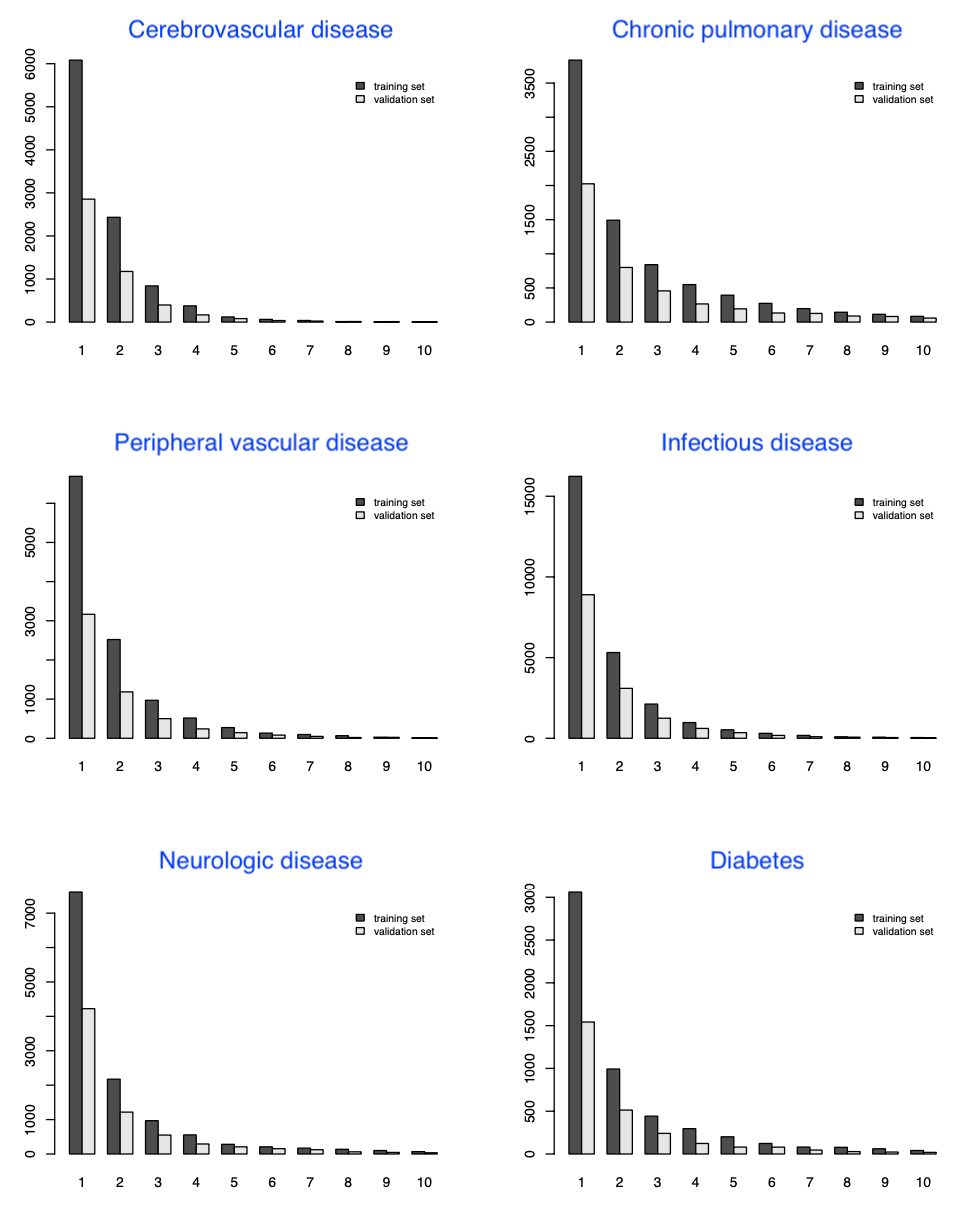


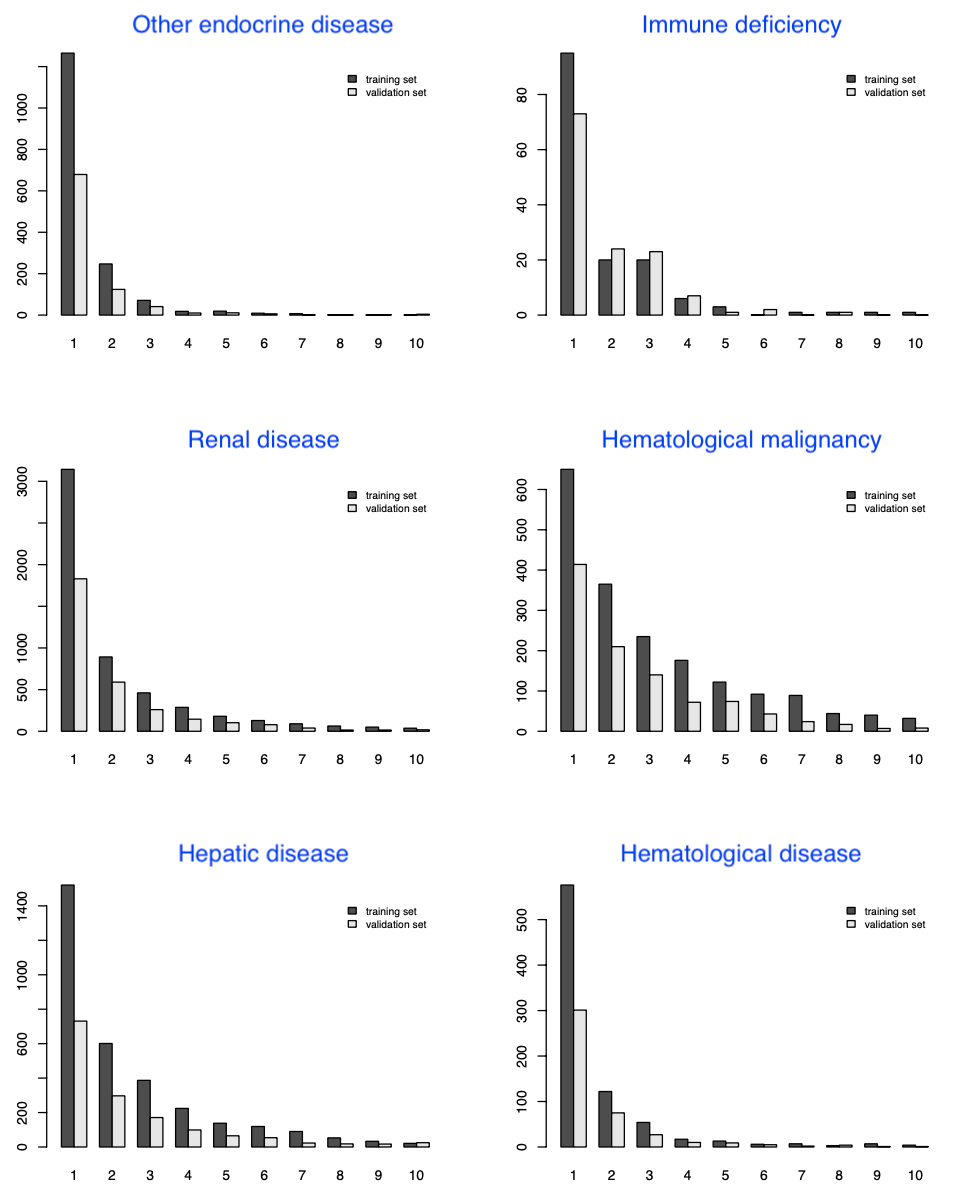


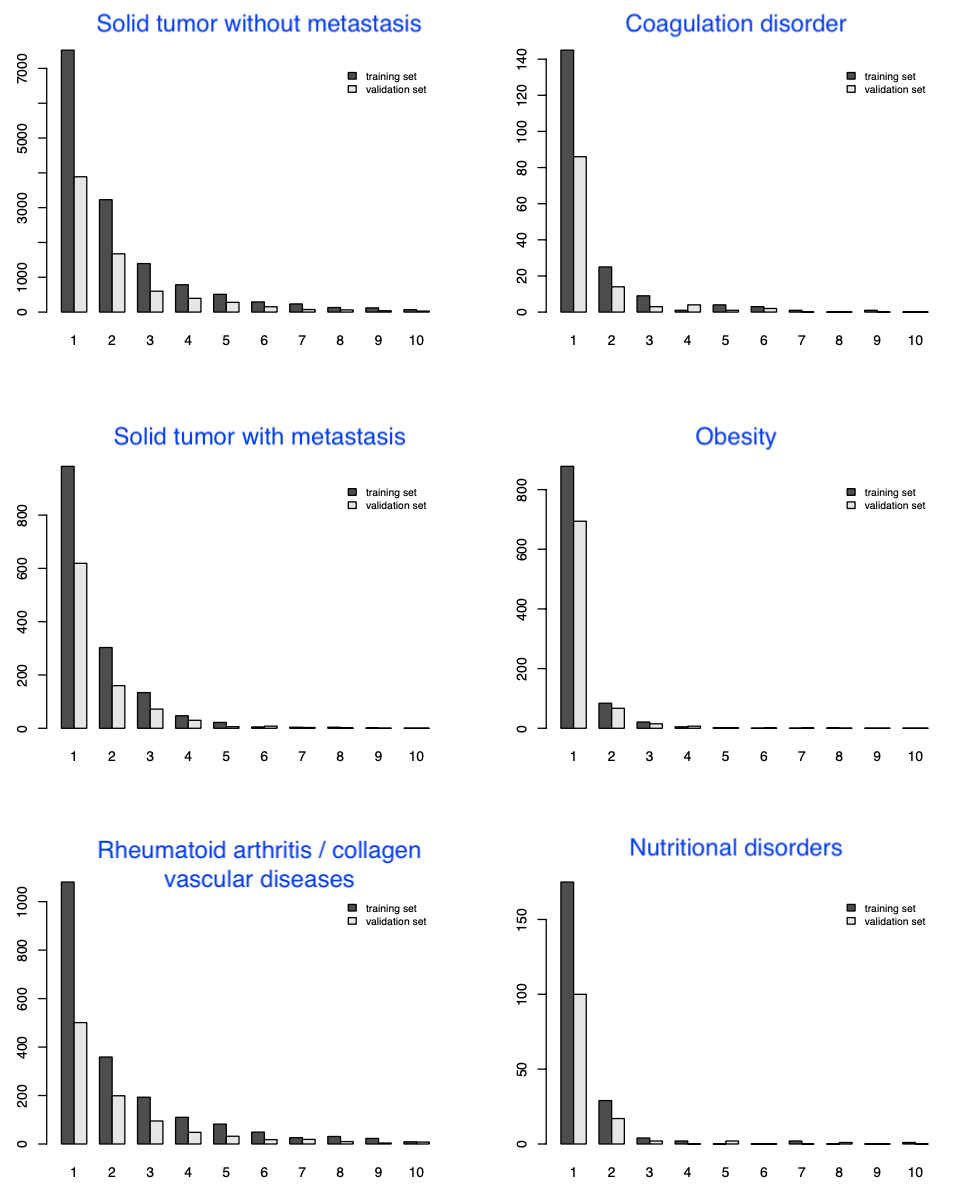


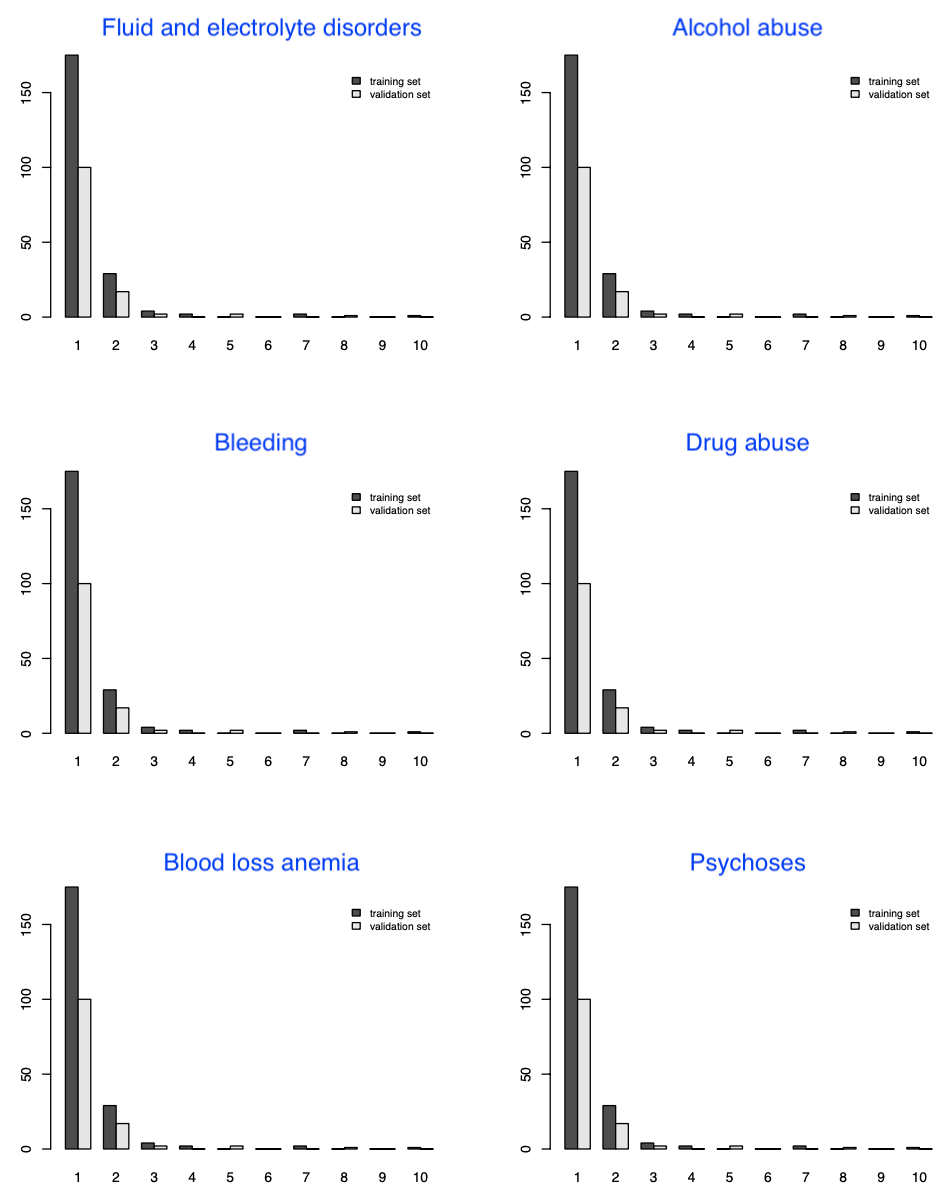


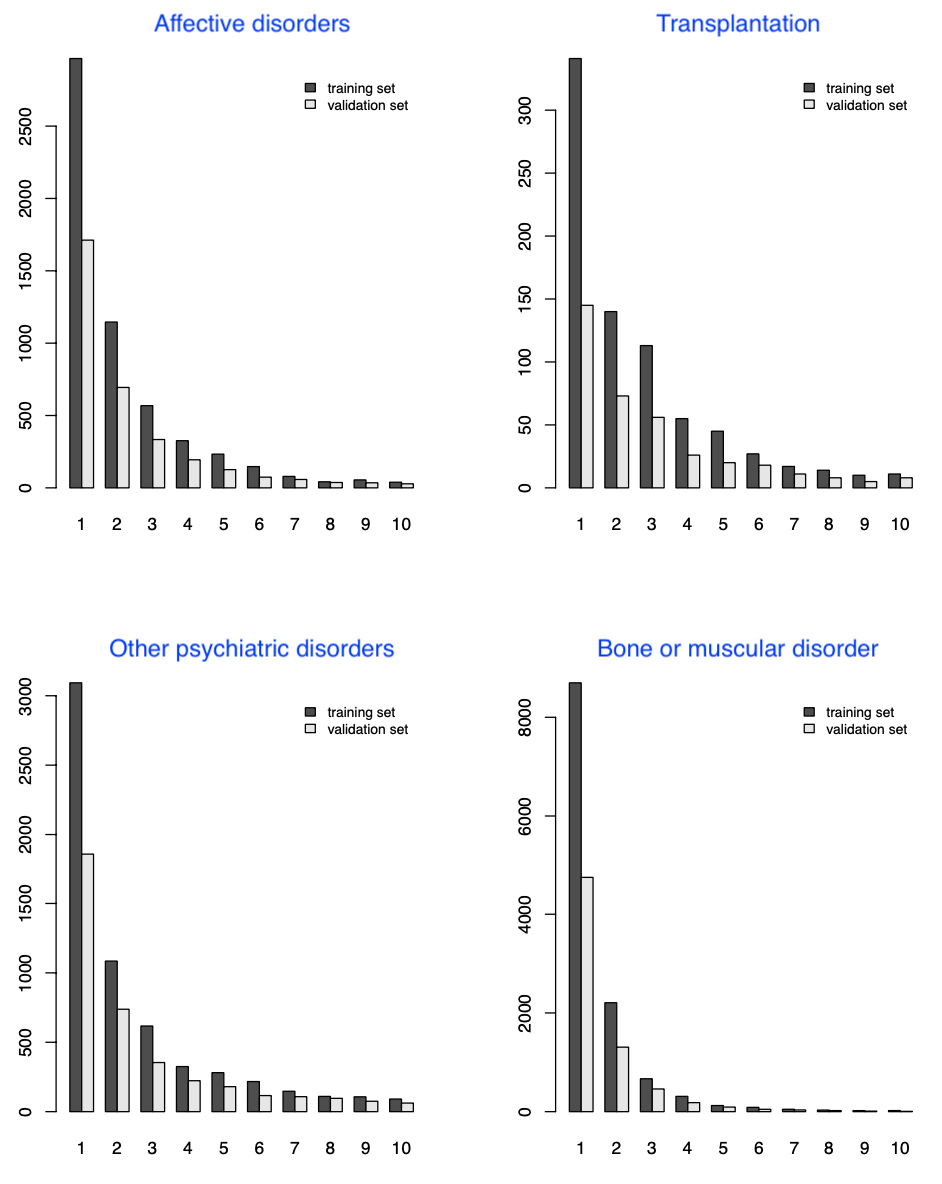


**eTable S2.** Variability in the number of previous hospital admissions with a principal diagnosis in the respective comorbidity category.

| Comorbidity category | Range | Mean | Median | N (%) with no hospital admission in the respective category |
| --- | --- | --- | --- | --- |
| Congestive heart failure (CHF) | (0,66) | 0.12 | 0 | 217374 (94.5%) |
| Ischemic heart disease (IHD) | (0,47) | 0.28 | 0 | 200327 (87.1%) |
| Valvular disease (Valv) | (0,20) | 0.1 | 0 | 217572 (94.6%) |
| Cardiac arrythmias (Ary) | (0,71) | 0.12 | 0 | 215715 (93.8%) |
| Pulmonary circulation disorders (Pulmcirc) | (0,31) | 0.02 | 0 | 226831 (98.6%) |
| Hypertension (HT) | (0,17) | 0.01 | 0 | 227434 (98.9%) |
| Cerebrovascular disease (CVD) | (0,17) | 0.11 | 0 | 215307 (93.6%) |
| Peripheral vascular disease (Perivasc) | (0,53) | 0.14 | 0 | 213270 (92.7%) |
| Neurologic disease (Neurol) | (0,59) | 0.21 | 0 | 210298 (91.4%) |
| Chronic pulmonary disease (CPD) | (0,143) | 0.22 | 0 | 217026 (94.3%) |
| Infectious disease (Inf) | (0,31) | 0.33 | 0 | 189288 (82.3%) |
| Diabetes (Diab) | (0,121) | 0.1 | 0 | 221667 (96.4%) |
| Other endocrine disorders (Xendo) | (0,15) | 0.02 | 0 | 227531 (98.9%) |
| Renal disease (Renal) | (0,128) | 0.08 | 0 | 221529 (96.3%) |
| Hepatic disease (Hepatic) | (0,33) | 0.06 | 0 | 225215 (97.9%) |
| Immune deficiencies incl. HIV (Immundef) | (0,18) | 0 | 0 | 229770 (99.9%) |
| Hematological malignancies (Hemomal) | (0,50) | 0.04 | 0 | 227069 (98.7%) |
| Other hematological disease (Hemodis) | (0,99) | 0.01 | 0 | 228807 (99.5%) |
| Solid tumour without metastases (tumournomet) | (0,68) | 0.22 | 0 | 208227 (90.5%) |
| Metastatic cancer (Tumourmet) | (0,11) | 0.02 | 0 | 227653 (99%) |
| Rheumatic disease (Collagen) | (0,51) | 0.03 | 0 | 227059 (98.7%) |
| Coagulopathy (Coag) | (0,12) | 0 | 0 | 229756 (99.9%) |
| Obesity (Obese) | (0,8) | 0.01 | 0 | 228278 (99.2%) |
| Nutritional deficiences (Nutr) | (0,10) | 0 | 0 | 229721 (99.9%) |
| Fluid and electrolyte disorders (Fluid) | (0,36) | 0.03 | 0 | 226274 (98.4%) |
| Blood loss and anemia (Bleed) | (0,12) | 0 | 0 | 229756 (99.9%) |
| Deficiency and other anemia (Anemia) | (0,41) | 0.03 | 0 | 225092 (97.8%) |
| Alcohol abuse (Alco) | (0,167) | 0.32 | 0 | 214964 (93.4%) |
| Drug abuse (Drug) | (0,61) | 0.13 | 0 | 221398 (96.2%) |
| Psychoses (Psycho) | (0,65) | 0.05 | 0 | 226821 (98.6%) |
| Affective disorders (Affect) | (0,67) | 0.1 | 0 | 220930 (96%) |
| Other psychiatric disorders (Xpsych) | (0,79) | 0.23 | 0 | 218838 (95.1%) |
| Transplantation (Tx) | (0,67) | 0.02 | 0 | 228851 (99.5%) |
| Bone or muscle disease (Bonemusc) | (0,35) | 0.14 | 0 | 210812 (91.6%) |
| Injury (Injury) | (0,57) | 0.27 | 0 | 194721 (84.6%) |
| Poisoning (Intox) | (0,44) | 0.16 | 0 | 216817 (94.2%) |

# Cox proportional hazards regression

Lack of information of previous ICU admission was not considered missing and no imputation was performed. The proportional hazards assumption for the Cox models was checked using plots of Schoenfeld’s residuals for each variable.

# Goodness of fit

Goodness of fit in the validation dataset was assessed by estimating the deviance difference of the respective model when refitted on the validation dataset [15]. The difference approximately follows a χ2 distribution with as many degrees of freedom as the number of parameters in the model. The comparison with the χ2-distribution is expected to be conservative.

# C-index

Because we assume proportional hazards in the Cox regression models the C-index will be the same, regardless of which length of follow-up is chosen.

# Calibration

The calibration slope was estimated in the validation data as the parameter from a model with the only covariate being the linear predictor $\sum\hat{\beta}_{i}x_{i}$ derived from applying the model derived from the training data. It should optimally be close to 1. Calibration plots were generated for survival at 10 days, 30 days, 90 days, 1 year, 1.5 years, and 2 years. For each centile of the estimated survival probabilities a non-parametric survival curve was fitted, taking censoring into account, with the estimated survival probability from the Kaplan-Meier as a measure of the observed probability. The midpoint of the predicted probability class was plotted against the observed probability.

# The Brier score

The Brier score measures the average squared difference between the true event status and the estimated predictive values at a certain point in time t*. In case of no censoring the empirical Brier Score can be calculated as [3]


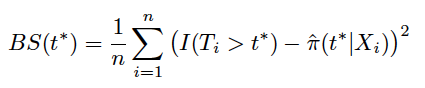


where I(T*_i_* > t*) = 1 if the observed time for individual *i* is greater than t*, 0 otherwise. The estimated survival probability for individual *i* with covariates X*_i_* is denoted by π(t*|X*_i_*). In case of censoring, there are three possibilities for everyone:

- If the survival time T*_i_* > t*, that is I(T*_i_* > t*) = 1 regardless of whether the individual has experienced the event at time T*_i_* or is censored and the contribution to the Brier score is (1 − π(t*|X*_i_*))^2^.
- If the survival time T*_i_* ≤ t* and the individual experienced the event at T*_i_* we know that the event status at t* is that it has occurred, hence *I*(T*_i_* > t*) = 0 and the contribution to the Brier score is (0 − π(t*|X*_i_*))^2^ = π(t*|X*_i_*))^2^.
- If the survival time T*_i_* ≤ t* and the individual was censored at T*_i_* we do not know the event status at time t* and hence cannot calculate the contribution to the Brier Score.

According to [3] the Brier Score can be calculated under random censorship by


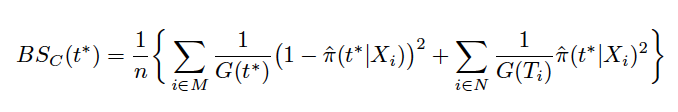


where M and N are the indices for the individuals with T*_i_* > t* and the individuals with T*_i_* ≤ t* and event respectively. The individuals with T*_i_* > t* are hence weighted with 1/G(t*) and the individuals in the other group are weighted with 1/G(T*_i_*) where G(*t*) is the Kaplan Meier estimate of *censoring* at time *t*. When the estimated probability is close to the true status, the difference will be small. The smaller Brier score the better.

**eTable S3.** Deviance difference calculated on the outcome death in the validation dataset. D.F is the deviance using the fitted parameters as fixed parameters, D.R the deviance when refitting the model on the validation dataset. The ­χ^2^_0.95_ is the 95^th^ percentile for the corresponding ­χ^2^ distribution. A deviance difference lower than this value indicates a good model fit. The deviance difference is therefore also presented as a proportion of the χ^2^_0.95_.

| Model | D.F | D.R | df | Deviance difference | χ^2^_0.95_ | Deviance difference as a proportion of χ^2^_0.95_ |
| --- | --- | --- | --- | --- | --- | --- |
| A | 340412.44 | 340345.87 | 12 | 66.57 | 21.03 | 3.17 |
| B | 338296.48 | 338169.53 | 18 | 126.95 | 28.87 | 4.40 |
| C0 | 337187.47 | 336891.88 | 48 | 295.59 | 65.17 | 4.54 |
| C | 338240.36 | 337924.4 | 48 | 315.96 | 65.17 | 4.85 |
| D | 338949.96 | 338335.82 | 48 | 614.14 | 65.17 | 9.42 |
| E | 338400.41 | 337679.55 | 84 | 720.86 | 106.39 | 6.78 |
| F | 336354.62 | 335769.65 | 228 | 584.97 | 264.22 | 2.21 |
| G | 336485.02 | 335936.16 | 192 | 548.86 | 225.33 | 2.44 |
| H | 336376.03 | 335776.64 | 228 | 599.39 | 264.22 | 2.27 |

**eFigure S8.** Cumulative incidence (Kaplan-Meier estimate) stratified according to risk groups defined by the linear predictor from the respective prediction model for the outcome death. The left panel is based on the most basic model (age + sex + number of ICU admissions + total length of previous ICU stays + time since last ICU discharge). The right panel is based on the selected final model H (…+ [Time since last hospital stay with principal diagnosis]_comorbidity category_ + [Length of hospital stay with principal diagnosis]_comorbidity category_). Black lines represent the training data set, and red lines the validation data set. The dashed horizontal blue lines indicate the increased separation of risk groups from using the selected final model with comorbidity indicated by the interval in days since discharge from the most recent hospital stay with a main diagnosis in the respective comorbidity category (Model H).

**
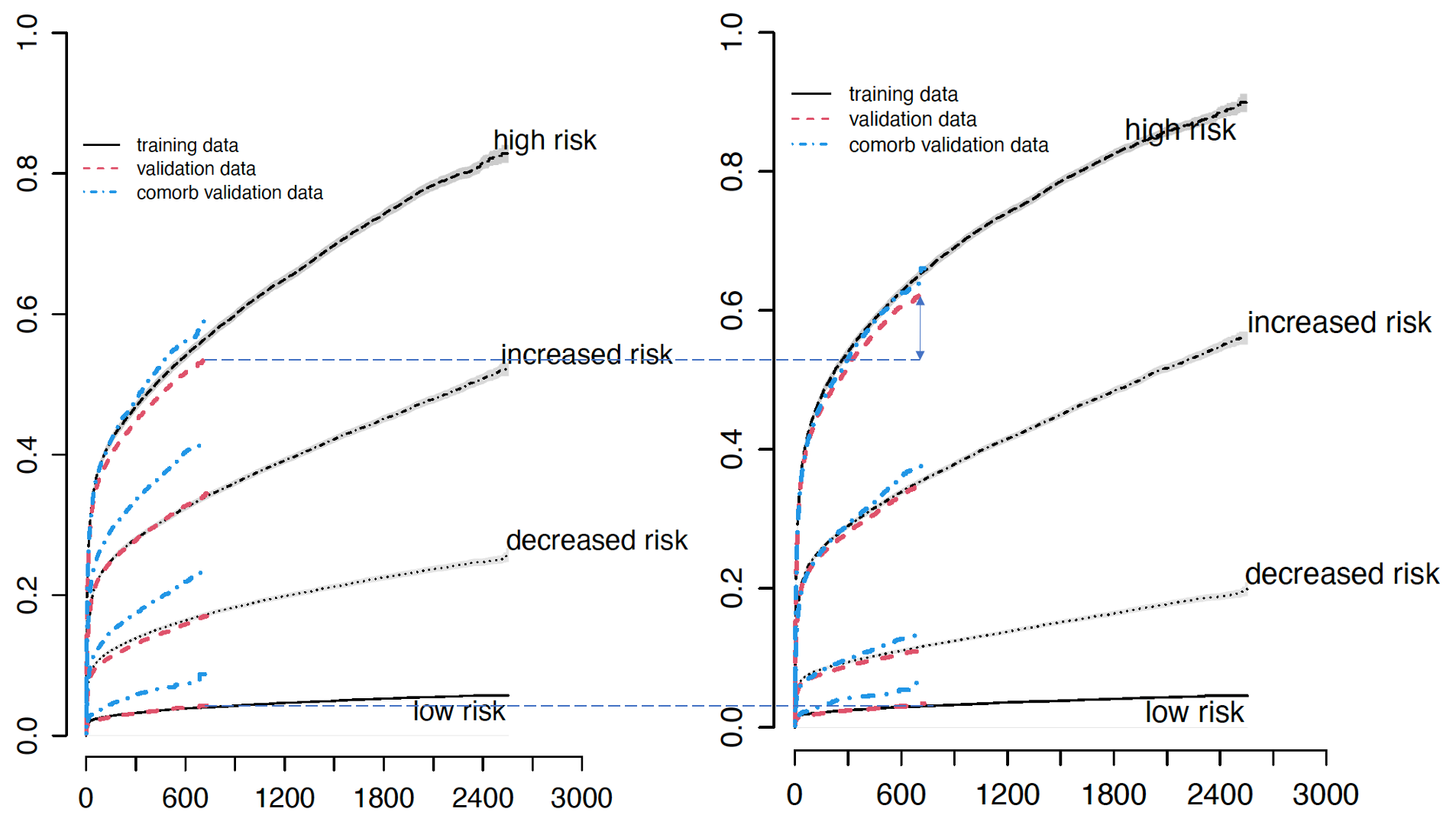
**

**eFigure S9.** Calibration plot for 10 days, 90 days, and 1 year event free period for the outcome death using the model A (Age + Sex + [Number of ICU admissions + Total length of ICU stays + Time since last ICU discharge]_365 days prior to the index admission date_) derived in the training dataset and applied to the overall validation dataset (left column), and the subgroup restricted to admissions where the patient had previous hospitalizations with main diagnoses representing at least two different comorbidity categories (right column). Gray vertical lines represent group borders of the centiles.


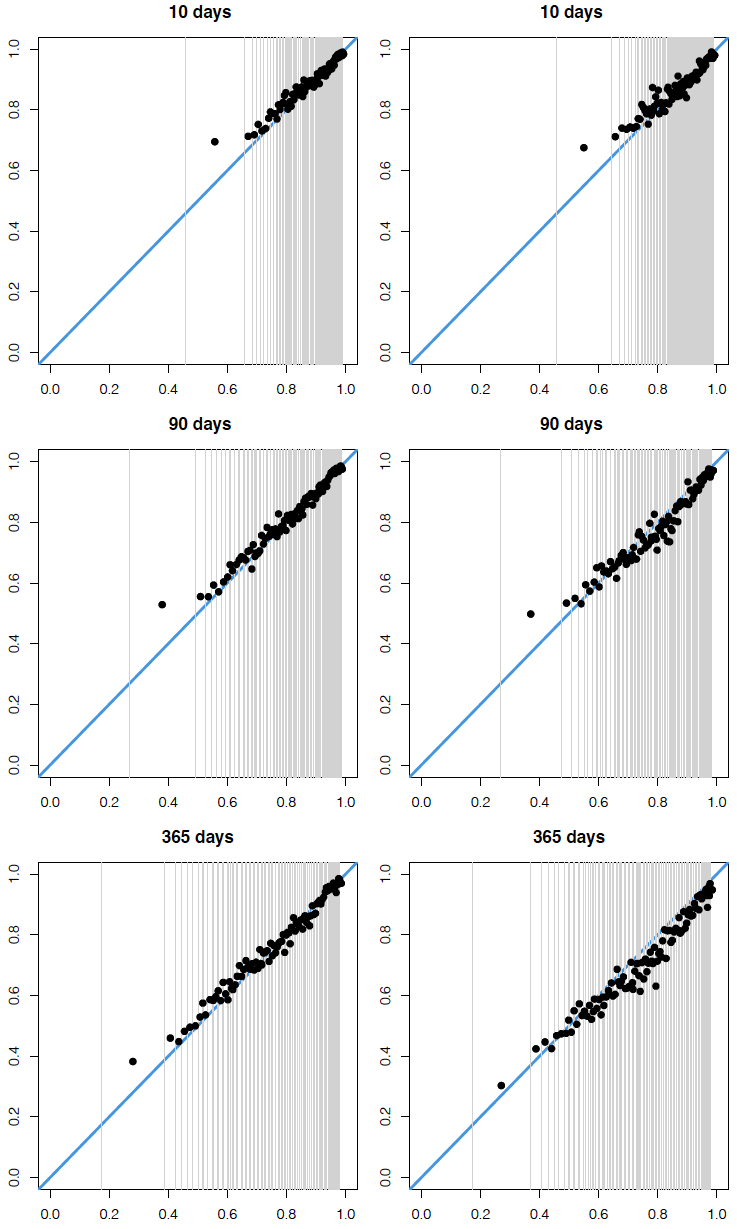


**eFigure S10.** Calibration plots for model based on Age + Sex + [*Variables indicating number of intensive care unit (ICU) admissions, total length of stay, and time since last ICU discharge during 365 days prior to the index admission date*] + [*Variables indicating the interval in days since discharge from the most recent hospital stay with a main diagnosis in the respective comorbidity category*] + [*Variables indicating the sum of length of hospital stays with a main diagnosis in the respective comorbidity category*]. Plots have been generated for 10 days, 90 days, and 1 year event free period for the outcome death in the training dataset and applied to the overall validation dataset (left column), and the subgroup restricted to admissions where the patient had previous hospitalizations with main diagnoses representing at least two different comorbidity categories (right column). Gray vertical lines represent group borders of the centiles.


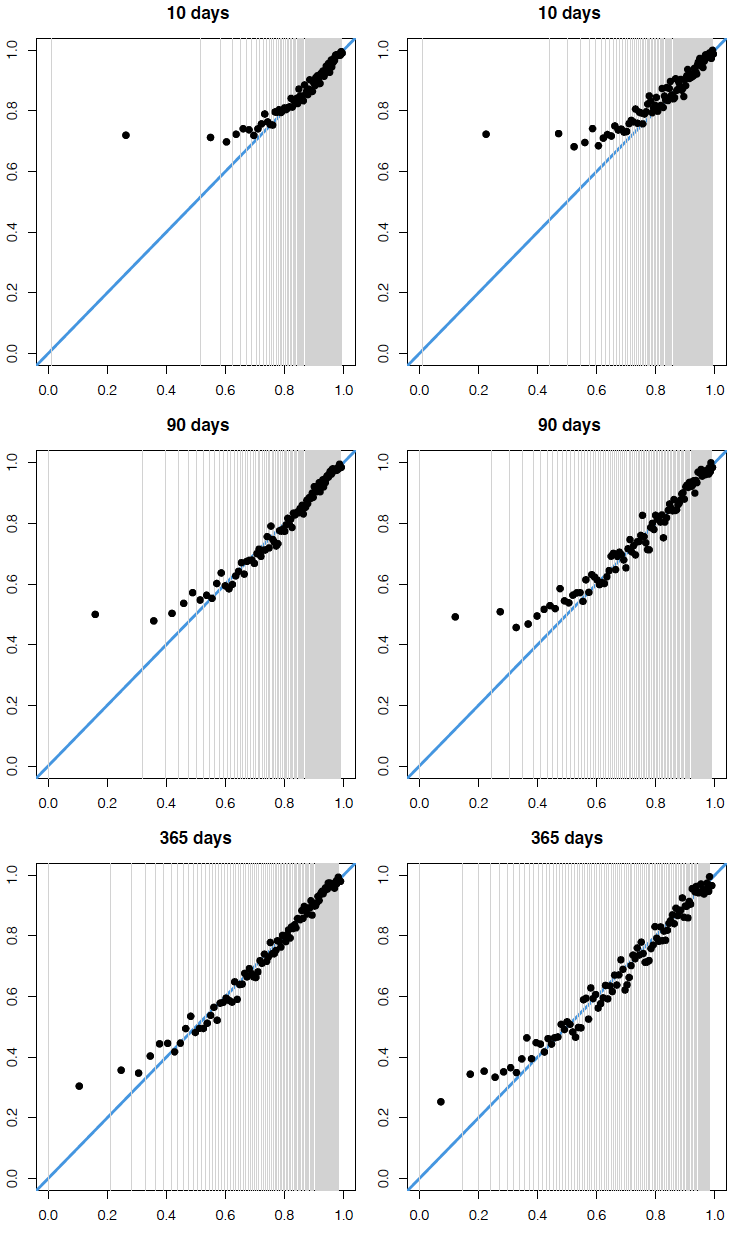


**eTable S4.** Brier score for different models and time points taking censoring into account by inverse probability censoring weighting. Results are presented for models developed in the training dataset and then applied to the validation dataset and the subgroup of the validation data with at least two different comorbidity categories.

|  | time points (days) | | | | |
| --- | --- | --- | --- | --- | --- |
|  | **10** | **30** | **90** | **180** | **365** |
| **Validation dataset** | | | | | |
| coxA | 0.09446 | 0.11742 | 0.13267 | 0.14284 | 0.15438 |
| coxB | 0.09479 | 0.11663 | 0.12994 | 0.13808 | 0.14692 |
| coxC0 | 0.09421 | 0.11550 | 0.12797 | 0.13572 | 0.14307 |
| coxC | 0.09397 | 0.11583 | 0.12912 | 0.13768 | 0.14630 |
| coxD | 0.09438 | 0.11614 | 0.12938 | 0.13791 | 0.14654 |
| coxE | 0.09424 | 0.11592 | 0.12882 | 0.13717 | 0.14548 |
| coxF | 0.09365 | 0.11434 | 0.12638 | 0.13378 | 0.14065 |
| coxG | 0.09371 | 0.11443 | 0.12657 | 0.13410 | 0.14109 |
| coxH | 0.09379 | 0.11446 | 0.12638 | 0.13382 | 0.14067 |
| **Subgroup of validation data with at least two different comorbidity categories** | | | | | |
| coxA | 0.11039 | 0.14010 | 0.16090 | 0.17565 | 0.19142 |
| coxB | 0.11070 | 0.13867 | 0.15703 | 0.16927 | 0.18185 |
| coxC0 | 0.11081 | 0.13815 | 0.15505 | 0.16655 | 0.17606 |
| coxC | 0.11025 | 0.13849 | 0.15641 | 0.16884 | 0.17955 |
| coxD | 0.11088 | 0.13874 | 0.15636 | 0.16868 | 0.17882 |
| coxE | 0.11073 | 0.13859 | 0.15584 | 0.16795 | 0.17817 |
| coxF | 0.10984 | 0.13613 | 0.15234 | 0.16333 | 0.17198 |
| coxG | 0.10992 | 0.13628 | 0.15264 | 0.16380 | 0.17276 |
| coxH | 0.11000 | 0.13621 | 0.15217 | 0.16317 | 0.17183 |

# The C-index

The *C-index* (and *Somers’ D_xy_* rank correlation, *D_xy_* = 2(*C* – $\frac{1}{2}$)) for a censored response variable can be calculated by considering all possible pairs of individuals, predict the probability of event-free time being larger than a specific time point and order the predicted probabilities and observed time to event within the pair. The *C*-index is the proportion of pairs where the rank of the predicted probabilities and the observed time of event is the same. Pairs where both subjects are censored or where one is censored with a follow-up time less than the uncensored one will be excluded. If the predicted probability is the same for the one with event and the one with a censored observation the pair contributes to the numerator with 0.5. *C* = 1 means a perfectly discriminating model, *C* = 0.5 for random ranks. We have calculated the *C*-index at a specific time point to calculate the event free probabilities but since we assume a proportional hazard model the *C*-index will be the same, regardless of which time point is chosen.

**eTable S5.** *C*-index for different models.

| Model | n | n events | n valid pairs | n concordant pairs | C-index |
| --- | --- | --- | --- | --- | --- |
| A | 76715 | 15941 | 964233891 | 682476702.0 | 0.707792 |
| B | 76715 | 15941 | 964233891 | 702159373.0 | 0.728204 |
| C0 | 76715 | 15941 | 964233891 | 716096054.5 | 0.742658 |
| C | 76715 | 15941 | 964233891 | 705379207.5 | 0.731544 |
| D | 76715 | 15941 | 964233891 | 703693654.5 | 0.729796 |
| E | 76715 | 15941 | 964233891 | 706964438.5 | 0.733188 |
| F | 76715 | 15941 | 964233891 | 722663719.5 | 0.749469 |
| G | 76715 | 15941 | 964233891 | 721678462.5 | 0.748448 |
| H | 76715 | 15941 | 964233891 | 721976837.5 | 0.748757 |

# Calibration

Calibration slope is the parameter from a model on the validation data set where the only covariate in the model is the linear predictor $\sum\hat{\beta}_{i}\chi_{i}$ calculated with the $\hat{\beta}_{i}$ from the original model on the training data set. With good calibration this slope should be close to 1.

**eTable S6:** Summary of calibration slope for different models for the outcome death.

| Model | Slope | 95% confidence interval |
| --- | --- | --- |
| A | 0.9234 | 0.902-0.945 |
| B | 0.9050 | 0.887-0.923 |
| C0 | 0.9092 | 0.892-0.926 |
| C | 0.9333 | 0.912-0.955 |
| D | 0.8670 | 0.820-0.914 |
| E | 0.8792 | 0.804-0.955 |
| F | 0.9107 | 0.893-0.928 |
| G | 0.9099 | 0.893-0.927 |
| H | 0.9129 | 0.896-0.930 |

**eTable S7:** Baseline characteristics for all admissions (*n* corresponds to number of admissions not unique individuals). The subgroup restricted to admissions where the patient had previous hospitalizations with main diagnoses representing at least two different comorbidity categories is presented.


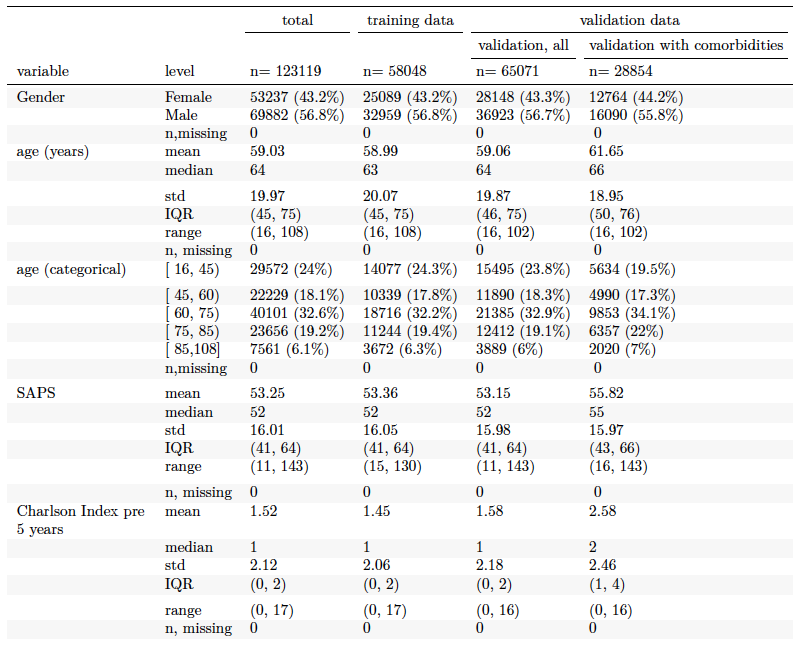


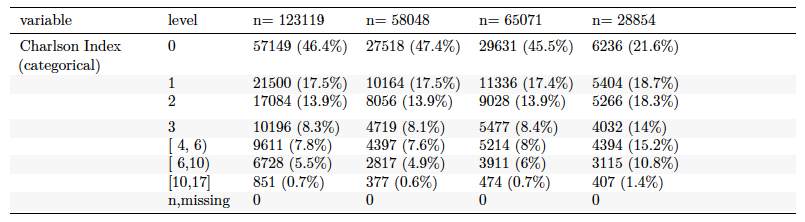


**eTable S8.** Comparison of different models’ ability to predict time to death in the training and validation datasets.

|  |  | **Akaike Information Criterion (AIC)** |  | **Deviance difference ^g^** | **Deviance difference**  $\boldsymbol{\chi}_{\boldsymbol{0.95}}^{\boldsymbol{2}}$ | **Brier score**  **(365 days)** | **C index** | **Calibration slope** |
| --- | --- | --- | --- | --- | --- | --- | --- | --- |
| **Model** |  | Training dataset |  | Validation dataset | | | | |
| A: Age + sex + *[Previous ICU]*^a^ |  | 1040287 |  | 66.57 | 21.03 | 0.154385 | 0.707792 | 0.9234 |
| B: A + Charlson Comorbidity Index |  | 1032953 |  | 126.95 | 28.87 | 0.146920 | 0.728204 | 0.9050 |
| C0: A + [Principal diagnosis Yes/No]_comorbidity category_^b^ |  | 1029370 |  | 295.59 | 65.17 | 0.143069 | 0.742658 | 0.9092 |
| C: A + [Count admissions with principal diagnosis]_comorbidity category_^c^ |  | 1033417 |  | 315.96 | 65.17 | 0.146299 | 0.731544 | 0.9333 |
| D: A + [Max count of admissions with principal or secondary diagnosis]_comorbidity category_^d^ |  | 1034706 |  | 614.14 | 65.17 | 0.146536 | 0.729796 | 0.8670 |
| E: C + [Length of hospital stay with principal diagnosis]_comorbidity category_^e^ |  | 1032622 |  | 720.86 | 106.39 | 0.145485 | 0.733188 | 0.8792 |
| F: C + [Time since last hospital stay with principal diagnosis]_comorbidity category_^f^ |  | 1026773 |  | 584.97 | 264.22 | 0.140645 | 0.749469 | 0.9107 |
| G: A + [Time since last hospital stay with principal diagnosis]_comorbidity category_^f^ |  | 1027101 |  | 548.86 | 225.33 | 0.141091 | 0.748448 | 0.9099 |
| H: A + [Time since last hospital stay with principal diagnosis]_comorbidity category_^f^ + [Length of hospital stay with principal diagnosis]_comorbidity category_^e^ |  | 1026880 |  | 599.39 | 264.22 | 0.140674 | 0.748757 | 0.9129 |

^a^ Variables indicating number of intensive care unit (ICU) admissions, total length of stay, and time since last ICU discharge during 365 days prior to the index admission date. ^b^ One variable for each comorbidity category, indicating the presence of at least one admission with a principal diagnosis from that category. ^c^ One variable for each comorbidity category, indicating the number of admissions with a principal diagnosis from that category. ^d^ One variable for each comorbidity category, indicating the number of admissions with a principal or secondary diagnosis from that category. ^e^ One variable for each comorbidity category, indicating the sum of length of hospital stays with a main diagnosis in that category. ^f^ One variable for each comorbidity category, indicating the interval in days since discharge from the most recent hospital stay with a main diagnosis in that category.

^g^ Deviance difference calculated on the validation data for the primary outcome death. D.F is the deviance using the fitted parameters as fixed parameters, D.R the deviance when refitting the model on the validation data. The χ20.95 is the 95th percentile for the corresponding χ2 distribution. A deviance difference lower than this value indicates a good model fit.

# SAPS score

A model with age, gender and SAPS score was fitted to the training data set and validated on the validation data set. From the General Additive Model (GAM) plot of SAPS score in eFigure S9 the SAPS score it was considered most appropriate to enter this variable in the model as two linear terms, SAPS and SAPS.50 where SAPS.50 is the extra slope after 50 defined as:


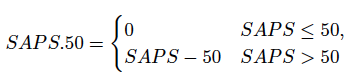


**eFigure S11:** GAM plot of SAPS score.


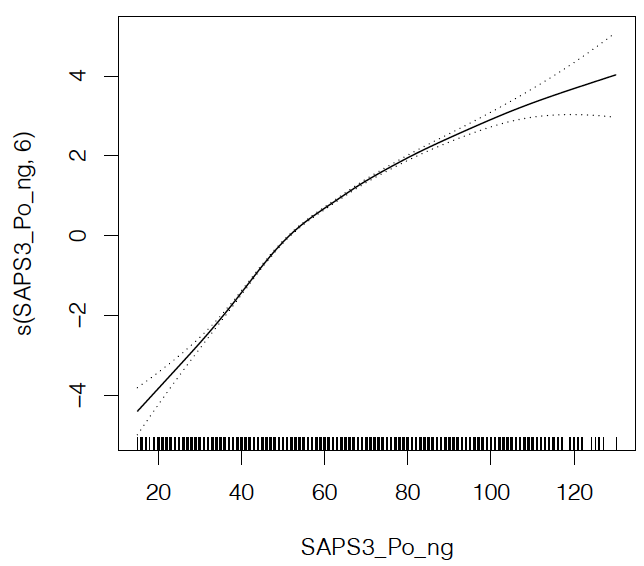


**eTable S9:** Brier score for a model with age, gender and SAPS score was fitted to the training data set and validated on the validation data set using different length of follow-up and taking censoring into account by inverse probability censoring weighting.


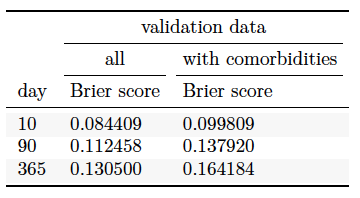


**eFigure S12.** Survival of patients in the age group 71-75 years presented separately for each subset defined by deciles of the baseline SAPS score. The survival probability is displayed stratified by quartiles of predicted probability of survival as measured by the linear predictor from a model with optimal selection of comorbidity variables (Model H; see Table 2 for description) but without age and sex as predictors in the model.


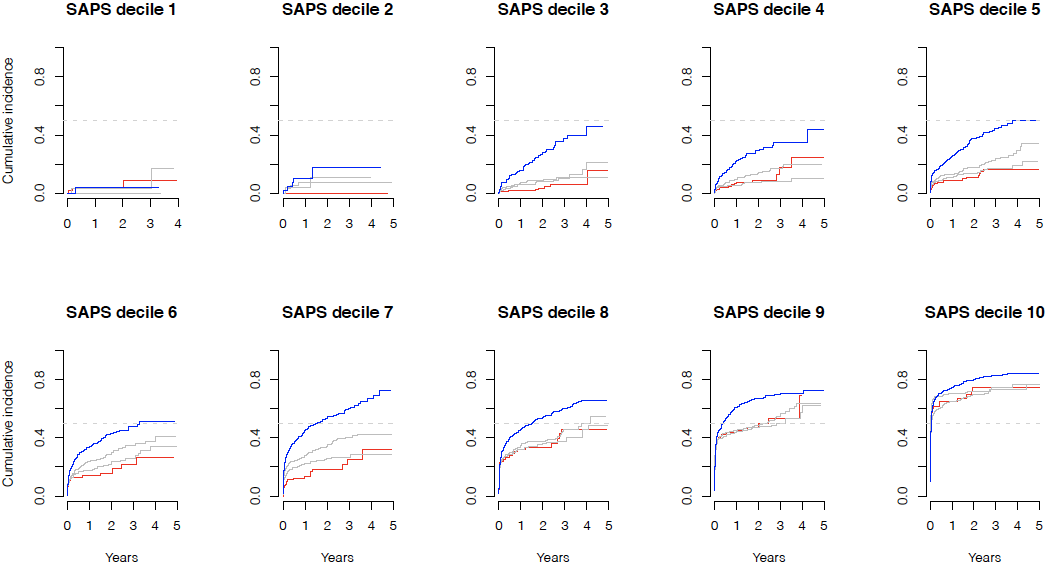


**eFigure S13.** Cumulative incidence of death and no readmission to the ICU plotted stratified by the linear predictor from applying model H (see Table 2 for description) was used as a summary measure of severity of comorbidity for each individual.


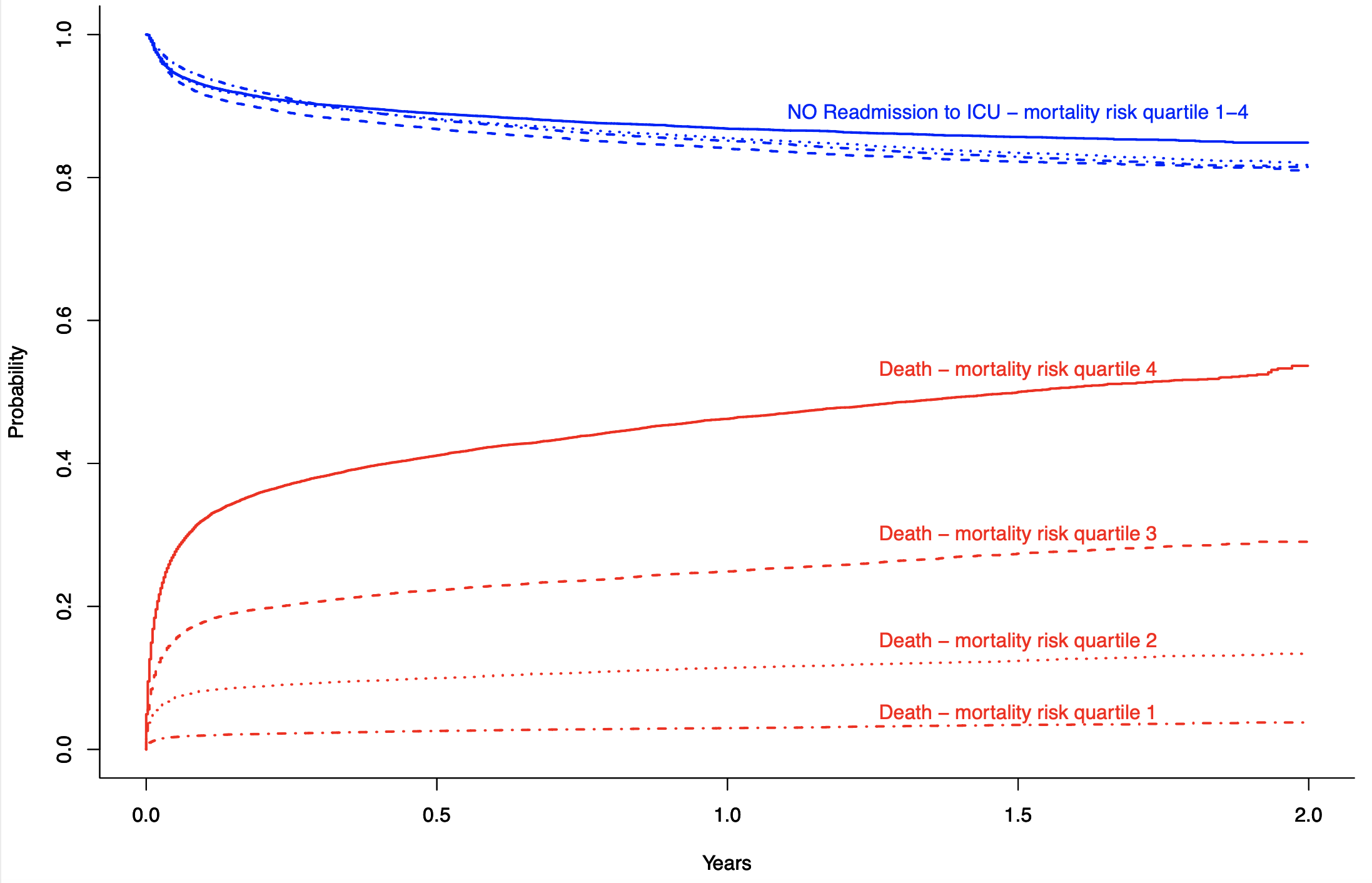

Supplement: Supplementary file 1 — Additional file 1. Optimized diagnosis-based comorbidity measures for all-cause mortality prediction in a national population-based ICU population - Supplementary online only material. [file 13054_2022_4172_MOESM1_ESM.docx]
